# Harnessing eukaryotic retroelement proteins for transgene insertion into human safe-harbor loci

**DOI:** 10.1038/s41587-024-02137-y

**Published:** 2024-02-20

**Authors:** Xiaozhu Zhang, Briana Van Treeck, Connor A. Horton, Jeremy J. R. McIntyre, Sarah M. Palm, Justin L. Shumate, Kathleen Collins

**Affiliations:** Department of Molecular and Cell Biology, University of California at Berkeley, Berkeley, CA USA

**Keywords:** Transposition, Molecular biology

## Abstract

Current approaches for inserting autonomous transgenes into the genome, such as CRISPR–Cas9 or virus-based strategies, have limitations including low efficiency and high risk of untargeted genome mutagenesis. Here, we describe precise RNA-mediated insertion of transgenes (PRINT), an approach for site-specifically primed reverse transcription that directs transgene synthesis directly into the genome at a multicopy safe-harbor locus. PRINT uses delivery of two in vitro transcribed RNAs: messenger RNA encoding avian R2 retroelement-protein and template RNA encoding a transgene of length validated up to 4 kb. The R2 protein coordinately recognizes the target site, nicks one strand at a precise location and primes complementary DNA synthesis for stable transgene insertion. With a cultured human primary cell line, over 50% of cells can gain several 2 kb transgenes, of which more than 50% are full-length. PRINT advantages include no extragenomic DNA, limiting risk of deleterious mutagenesis and innate immune responses, and the relatively low cost, rapid production and scalability of RNA-only delivery.

## Abstract (web summary)

Transgenes are inserted into human cells by 2-RNA delivery of a retroelement protein and template.

## Main

**Fig. 1:**
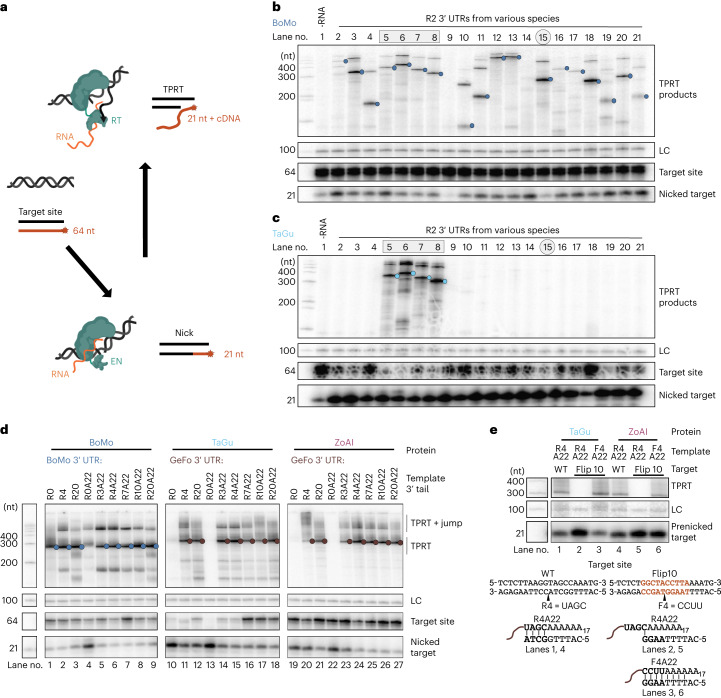
Biochemical activities and specificities of avian R2 proteins. **a**, Schematic of TPRT assay using target-site duplex with radiolabeled 5′ end indicated by a star. Created with BioRender.com. **b**–**e**, TPRT assays. For all TPRT panels including **b**–**e**, denaturing PAGE resolution of reaction products was done on a single gel with different size ranges cropped. In **b**–**d**, full-length TPRT products from copying a single template are denoted by colored circles, whereas TPRT + jump products extend the initial cDNA. LC is the loading normalization control added before product precipitation. **b**,**c**, R2 proteins BoMo (**b**) and TaGu (**c**) were tested for their ability to use R2 3′ UTR RNAs from different species as TPRT templates, each with an R4 3′ tail. R2 3′ UTR used is as follows: 1, no template; 2, *Hydra magnipapillata*; 3, *Adineta vaga*; 4, *Limulus polyphemus*; 5, *Zonotrichia albicollis*; 6, *Tinamus guttatus*; 7, *Taeniopygia guttata*; 8, *Geospiza fortis*; 9, *Gasterosteus aculaetus*; 10, *Oryzias latipes*; 11, *Pungitius pungitius*; 12, *Tribolium castaneum*; 13, *Nasonia vitripennis*; 14, *Ciona intestinalis*; 15, *Bombyx mori*; 16, *Lepidurus couesii*; 17, *Triops cancriformis*; 18, *Drosophila simulans*; 19, *Drosophila mercatorum*; 20, *Drosophila melanogaster*; and 21, *Drosophila nasuta*. Lane with *B. mori* 3′ UTR is highlighted with a circled number; lanes with avian 3′ UTR are highlighted with a box around the numbers. **d**, Comparison of different template 3′ tails. **e**, The top shows the TPRT assay using prenicked target sites with GeFo 3′ UTR template containing R4A22 or F4A22 3′ tail. The bottom shows a schematic of 10 bp reverse-complement change in Flip10 and the corresponding template R4 or F4 3′ tail base pairing.

Gene therapy approaches are constantly optimized for their application to human disease. While engineered CRISPR–Cas systems excel in gene disruption and nucleotide correction, their use for transgene insertion by DNA break repair has limitations^1^. Viral vector strategies can achieve non-replicating episomal or randomly integrated transgene delivery but with high risk of immune response and/or genome mutagenesis^2^. Ideally, therapeutic transgenes of choice could be stably introduced to the human genome at a safe-harbor locus. Safe-harbor genome insertion has been favored in eukaryotic evolution by site-specific retroelements^3^. Some non-long terminal repeat (non-LTR) retroelements show exquisite insertion-site specificity beneficial for safeguarding the host genome against insertional mutagenesis^4^. Loss of this specificity, for example by the human LINE-1 retroelement, makes retroelement silencing essential for genome stability and function^5^. Because non-LTR retroelement insertion uses an RNA template for new gene synthesis, typically with the transcript 3′ untranslated region (UTR) recognized by the reverse transcriptase (RT) protein, no donor DNA is involved. Additionally, because non-LTR retroelements insert by complementary DNA (cDNA) synthesis directly into the genome using target-primed reverse transcription (TPRT, Fig. 1a), there is not any stage of extrachromosomal DNA to trigger an innate immune response^6^. Furthermore, retroelement-protein endonuclease domain (EN) nicking of target-site strands is sequential, with second-strand nicking activated by first-strand synthesis^7^. Thus, the nick that initiates second-strand synthesis would not generate blunt duplex ends prone to mutagenic re-ligation by canonical nonhomologous end joining without gene insertion. Despite these possible advantages of retroelement-protein synthesis of a transgene, there are potential challenges as well. For example, previous attempts to insert a transgene encoded separately from active protein (in *trans*) reveal that this is much less efficient than mobility of a native non-LTR retroelement^8–10^, potentially due to the ‘*cis* preference’ of newly synthesized protein assembly with its own encoding RNA^11^. Also, verification of bona fide *trans*-templated transgene insertions requires the detection of a 5′ junction as well as a 3′ junction, to indicate that second-strand synthesis has occurred.

Here, we overcome the challenges to adapt non-LTR retroelement machinery for gene addition to the human genome using target-site-specific members of the R2 retroelement family, which harbor a single open reading frame (ORF). R2 is detected in the genomes of diverse metazoans^12^. Mammals lost R2 elements but retain the conserved target site. R2 inserts within the multicopy ribosomal RNA (rRNA) gene locus (rDNA) transcribed by RNA polymerase (RNAP) I^7^. Sequence-specific insertion can be recapitulated in vitro using purified protein, RNA and genomic DNA (gDNA)^13^. N-terminal zinc finger and Myb DNA-binding domains are the primary determinants of target-site recognition^14,15^. This has been very recently visualized by cryogenic-electron microscopy^16,17^ of the D-clade R2 protein from *Bombyx mori* (hereafter, BoMo), the only R2 protein previously purified and shown to have TPRT activity^7^. Because the target sequence is in a gene present in hundreds of copies per genome, which in human cells are in tandem arrays that constitute the short arms of five acrocentric chromosomes^18^, cell function is not compromised by retroelement-insertion-mediated inactivation of a few, or in some organisms at least half, of the rDNA units^19,20^. Although rDNA arrays are prone to restructuring in meiosis, cancers or cells with specific DNA repair deficiencies, they are mitotically stable in normal human somatic tissues^21–23^. Long-term expression of transgenes integrated into rDNA has been demonstrated using several strategies of donor DNA delivery and integration. Therapeutic proteins are expressed from rDNA-integrated transgenes in mice and human cells, including, for example, blood clotting cascade Factors VIII and IX (deficient in Hemophilia A and B), fumarylacetoacetate hydrolase (deficient in tyrosinemia type I) and mini-dystrophin^24–33^. These precedents encouraged us to exploit R2 retroelement insertion specificity as the starting point for developing PRINT.

We developed a method for stable, safe transgene supplementation of the human genome by delivery of RNA. An RNA-based approach can minimize deleterious immune responses and protect against random genome insertions arising from extragenomic DNA. Our adaptation of a eukaryotic retroelement protein with highly coordinated RNA and DNA binding, nicking and cDNA synthesis activities gives PRINT a high specificity for template RNA and target DNA even before engineering improvements.

## Results

### Template selectivity of retroelement proteins

**Extended Data Fig. 1:**
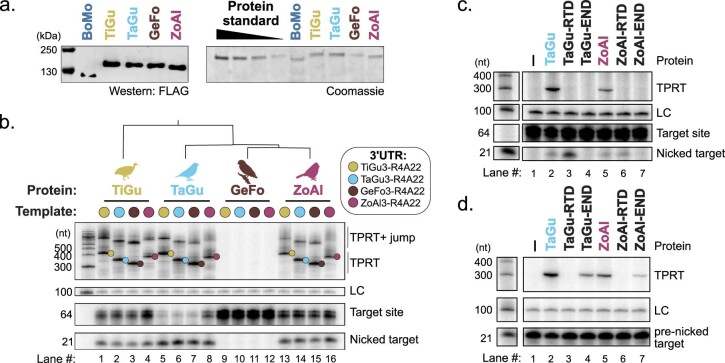
R2 protein purification and activities. **a**, Immunoblot and Coomassie stain of affinity purified R2 proteins resolved by SDS-PAGE. Coomassie staining confirmed the purity of R2 proteins and concentration by comparison to a titration of protein standard. **b**, TPRT assay of 4 avian R2 proteins with each of the 4 avian R2 3’UTRs. Templates contained the indicated 3’UTR followed by 4 nt rRNA (R4) and an A-tract (A22). **c-d**, Functional analysis of RTD and END versions of TaGu and ZoAl proteins. Template RNA had the GeFo 3’UTR and R4A22 tail. Oligonucleotides were annealed to generate an intact target site in **c** or a pre-cleaved target site in **d**.

We screened for RT and EN biochemical activities across previously inventoried and newly reconstructed A- and D-clade R2 retroelement ORFs, each codon-optimized, N-terminally FLAG-tagged and transiently expressed in and purified from human embryonic kidney 293T (HEK293T) cells (Extended Data Fig. 1a). Each R2 protein was combined with each of a large panel of potential template RNAs (Supplementary Table 1a), including diverse species’ R2 3′ UTRs with divergent length, sequence and predicted secondary structure. The ribonucleoprotein combinations were tested for sequence-specific target-site nicking and efficient use of nicked primer for cDNA synthesis using DNA oligonucleotide duplexes (Supplementary Table 1b) with a 5′ radiolabel on the primer strand (Fig. 1a). TPRT assays included the D2-clade BoMo, which as expected showed precise target-site cleavage and productive TPRT; however, its RNA template choice was extremely promiscuous in that nearly all tested templates were used for TPRT (Fig. 1b). By contrast, A3-clade proteins from avian species had not only precise target-site cleavage and productive TPRT but also high template selectivity for only avian R2 3′ UTRs (Fig. 1c). All R2 proteins generated TPRT product by copying a single template RNA and also made longer products by template jumping from full-length cDNA to additional molecules of 3′ UTR RNA that are in excess in the reconstituted reaction^34^. R2 proteins from *Taeniopygia guttata* (zebrafinch, TaGu), *Zonotrichia albicollis* (white-throated sparrow, ZoAl) and *Tinamus guttatus* (tinamou, TiGu) but not *Geospiza fortis* (medium ground finch, GeFo) were biochemically active for TPRT using avian R2 3′ UTR templates (Extended Data Fig. 1b). TPRT activity was eliminated by side chain substitution in the RT or EN active site, but RT-dead (RTD) proteins retained EN cleavage activity and EN-dead (END) proteins supported cDNA synthesis at a prenicked target site (Extended Data Fig. 1c,d).

In the original studies of bacterially expressed BoMo protein, TPRT activity was optimal using BoMo 3′ UTR template ending precisely at the retroelement–rDNA boundary, with no downstream ribosomal RNA (rRNA)^35^. In our assays as well, using recombinant protein purified from human cells, BoMo used its 3′ UTR as template with or without a 3′ tail of primer-complementary rRNA following the UTR (Fig. 1d, lanes 1–9). By contrast, TPRT by avian R2 proteins required the template to possess a 3′ tail with rRNA sequence immediately downstream from the target-site nick that could base pair with the primer (Fig. 1d, lanes 10–27). A length of 4 nucleotides (nt) of rRNA (R4) was sufficient, and cDNA synthesis from the nick was improved by appending a terminal tract of 22 adenosines (A22) following R4 (Fig. 1d, lanes 10–27). TPRT product length did not change with rRNA tail lengths from 3 to 20 nt (R3 to R20), with or without the addition of A22, suggesting that this 3′ tail portion of template was not copied into cDNA.

To establish the importance of template RNA base pairing with primer, 10 base pairs (bp) of target-site sequence surrounding the wild-type (WT) nick site was changed to its reverse complement (Flip10) and the R4 sequence of template 3′ tail was changed to match the mutant target site (F4), generating up to 7 bp of primer-template pairing (schematics in Fig. 1e). The Flip10 sequence change impaired nicking, so we used prenicked target-site duplexes to test the influence of template 3′ tail sequence on TPRT. Template RNA with 3′ tail R4A22 was used by both TaGu and ZoAl at the WT but not Flip10 target site (Fig. 1e, lanes 1–2 and 4–5). TPRT could be rescued at the Flip10 target site by changing the template RNA R4 to base pair with Flip10 prenicked primer (F4A22; Fig. 1e, lanes 3 and 6). We conclude that avian A-clade R2 proteins have a more stringent requirement for DNA–RNA base pairing immediately downstream of the cleavage site than the D-clade BoMo R2 protein, adding a second layer of target-site specificity to the extensive upstream DNA recognition by N-terminal DNA-binding domains^15–17^.

### Transgene insertion in human cells

**Extended Data Fig. 2:**
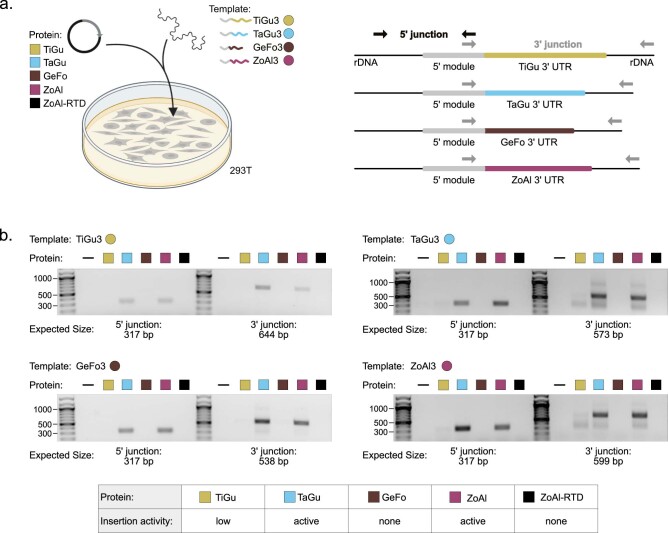
Transgene insertion by sequential transfection of protein-encoding plasmid and template RNA. HEK293T cells were transfected with plasmid encoding R2 protein, then 2 days later transfected with template RNA. Cells were harvested 1 day after template transfection. ZoAl-RTD was used as a negative control. **a**, Illustration of assays using plasmid-encoded protein and template RNA. HEK293T cells were used for high serial transfection efficiency. Created with BioRender.com. At right, transgene insertions are depicted with PCR primer positions indicated. **b**, PCR detection of 5’ and 3’ transgene junctions in rDNA, with summary of results below.

The specificity of avian R2 proteins for the avian R2 3′ UTR, and their requirement for template 3′ tail base pairing with a nicked target site, suggested them as candidates for achieving template-selective TPRT in human cells. Early assays using plasmids to express template RNA in cells revealed false positives of DNA-templated transgene insertion and transgene 5′ junction products generated during PCR from the rDNA sequence overlap of plasmid and target site. Therefore, we transfected purified, in vitro transcribed (IVT) template RNA and plasmid encoding an R2 protein into HEK293T cells (Extended Data Fig. 2a). Template RNAs contained a 5′ module derived from the 5′ end of the B-clade R2 retroelement in *Tribolium castaneum*, which was the shortest hepatitis delta virus-like native R2 ribozyme^36,37^ that we confirmed to be active for self-cleavage and thereby knew had adopted structure during IVT. The double-pseudoknot tertiary structure of a hepatitis delta virus ribozyme fold has exceptional resistance to degradation by exonucleases, affording a long half-life in cells^38^. In the template RNA context, self-cleavage generates a 5′ end 28 nt of rRNA, which as cDNA could base pair upstream of the target-site nick. One day after transfection of template RNA, gDNA was purified for PCR assays of transgene insertion. PCR readily detected transgene insertion at the target site using TaGu or ZoAl protein, with much lower insertion activity detected for TiGu and none detected for GeFo (Extended Data Fig. 2b). We detected not only 3′ transgene junctions but also 5′ transgene junctions indicative of double-stranded transgene insertion embedding transgene DNA at the rDNA target site (Extended Data Fig. 2b).

**Fig. 2:**
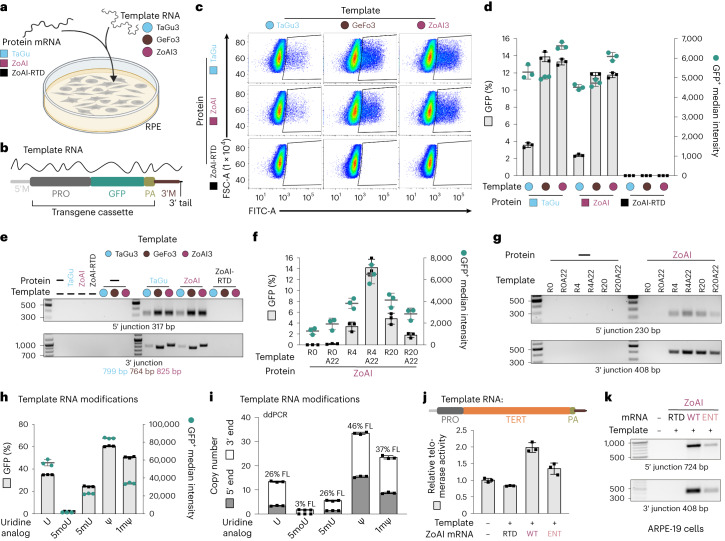
Transgene insertion by separate protein mRNA and template RNA. **a**, Schematic of 2-RNA transfection. Created with BioRender.com. **b**, Schematic of template RNA. M, module. **c**, Flow data for a representative replicate of data graphed in **d**, with forward scatter (FSC-A) on the *y* axis indicating measurement of cell size and fluorescein isothiocyanate (FITC-A) on the *x* axis indicating measurement of GFP intensity. **d**, Bar graph of %GFP^+^ cells and their median GFP intensity comparing templates with TaGu, GeFo or ZoAl 3′ UTR. **e**, PCR detection of 5′ and 3′ transgene junctions from a representative replicate of the experiment in **d**. Here and subsequently, expected PCR product sizes are given. **f**, Bar graph of %GFP^+^ cells and their median GFP intensity comparing transgene insertions from GeFo 3′ UTR templates with different 3′ tails indicated. **g**, PCR detection of 5′ and 3′ transgene junctions in a representative replicate of the experiment in **f**. **h**, Bar graph of %GFP^+^ cells (left *y* axis) and their median GFP intensity (right *y* axis) comparing transgene insertions from GeFo 3′ UTR_R4A22 templates with varying uridine modification. **i**, ddPCR measurement of average insertion copy number and the percentage full-length transgene from the transfected cell pools in **h**. The white bar starts from the *x* axis, not from the top of the gray bar. **j**, Quantitative PCR assay for telomerase activity in cells with transgenes generated using the template RNA schematized at top. ZoAl-ENT has tempered endonuclease activity (below). **k**, PCR detection of TERT transgene insertion junctions. In any relevant panel, data are presented as mean values ± error bars indicating standard deviation for three technical replicates.

Transgene insertion was next accomplished by cotransfecting template RNA with messenger RNA (mRNA) encoding R2 protein (Fig. 2a, 2-RNA system). As a cell culture system, we used human hTERT RPE-1 cells (hereafter RPE), a nontransformed human cell line originating from retinal pigment epithelium^39^. We used templates encoding an expression cassette for green fluorescent protein (GFP) (Fig. 2b) and quantified efficiency of transgene insertion by comparing the percentage of cells expressing GFP (%GFP^+^) and median GFP intensity of GFP^+^ cells using flow cytometry (hereafter flow). Cells were also collected for gDNA purification to detect transgene insertions by PCR. For consistency across experiments, unless noted otherwise, flow and insertion-junction PCR used cells gathered 20–24 h after transfection (see below for the time course). TaGu and ZoAl R2 proteins gave the highest %GFP^+^ cell counts and junction PCR signals using templates with GeFo or ZoAl 3′ UTR, with reduced efficiency using TaGu 3′ UTR even with TaGu protein (Fig. 2c–e). GFP signal was dependent on transgene insertion by TPRT: negative controls included template RNA alone and the more stringent negative control of template RNA cotransfected with RTD protein (Fig. 2c–e). These controls rule out transgene sequence insertion by RNA-templated host-cell repair pathways^40,41^, which could be activated by an R2 protein EN-mediated target-site nick or break.

**Extended Data Fig. 3:**
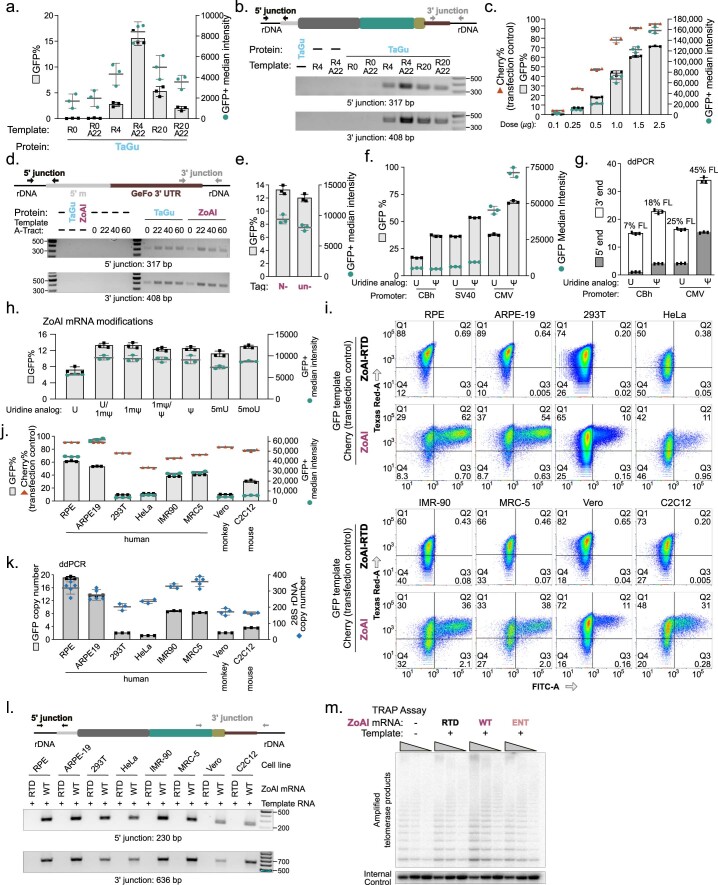
Transgene insertion by 2-RNA delivery. **a-b**, Comparison of TaGu- mediated transgene insertion using templates with different 3’ tails indicated. **a**, Bar graph of %GFP+ cells and their median GFP intensity. **b**, PCR detection of 5’ and 3’ transgene junctions from a representative replicate of cells in **a**. **c**, RNA dose influence on %GFP+ cells and their median GFP intensity, using ZoAl mRNA. **d**, PCR detection of transgene insertion junctions for templates varying in the number of terminal adenosines. **e**, mRNAs encoding untagged or N-terminally FLAG-tagged ZoAl were tested in parallel. **f**, Transgene GFP expression from CBh, SV40, and CMV promoters was compared following 2-RNA delivery of untagged ZoAl mRNA and template RNA made with uridine or ψ. **g**, ddPCR results for a representative replicate of total transfected cell pools from **f**. Note that the white bar starts from the x-axis, not from the top of the gray bar. **h**, Comparison of mRNA transcripts with different uridine nucleotides. Any mixture indicated was equimolar. **i-l**, PRINT insertions in various cell lines. **i**, Flow plots for a representative replicate of the data graphed in **j**. Texas Red-A on the y-axis is a measurement of mCherry expression (transfection control) and FITC-A on the x-axis is a measurement of GFP expression. Each cell line was assayed in parallel with ZoAl-RTD negative control (above) or ZoAl (below) mRNA. **j**, Bar graph of average %GFP+ cells and their median GFP intensity. ZoAl mRNA and template RNA were mixed at 1:6 molar ratio and transfection used 1.5 μg RNA with 4.5 μL transfection reagent. **k**, ddPCR to detect copy number for the GFP ORF from total transfected cell pools and 28 S rDNA copy number from untransfected cells per genome. **l**, PCR detection of insertion junctions. **m**, see main text for definition of ENT. Two-step telomerase activity assay using primer extension followed by PCR, with products radiolabeled by nucleotide incorporation and resolved by denaturing PAGE. TRAP, telomeric repeat amplification protocol. Extract titrations are 3-fold dilutions. In any relevant panel, data are presented as mean values +/- error bars indicating standard deviation for 3 technical replicates.

To further optimize transgene insertion, various features were interrogated. Matching TPRT results in vitro, the template RNA 3′ tail configuration R4A22 gave greater %GFP^+^ cells and rDNA insertion-junction PCR signals than no rRNA (R0 or R0A22) or a longer length of rRNA sequence (R20 or R20A22) (Fig. 2f,g for ZoAl and Extended Data Fig. 3a,b for TaGu). Increase in %GFP^+^ cells by the R4A22 3′ tail, or by increased RNA delivery dose, was accompanied by an increase of median GFP intensity (right side *y* axes in Fig. 2f and Extended Data Fig. 3a,c), resulting at least in part from an increase in transgene copy number per cell (below). Use of longer A-tracts did not increase insertion efficiency (Extended Data Fig. 3d). N-terminally tagged and untagged R2 protein gave comparable results (Extended Data Fig. 3e). Among the transgene promoters tested, in addition to the initial nonviral CBh promoter, templates with versions of Simian Virus 40 (SV40) and cytomegalovirus (CMV) immediate–early promoters were used efficiently for transgene synthesis and gave strong GFP expression (Extended Data Fig. 3f,g).

Relevant to delivery of a 2-RNA system as gene therapy, the mRNA could be fully substituted with base-modified uridines including pseudouridine (ψ), N1-methylpseudouridine (1mψ), 5-methoxyuridine (5moU) or 5-methyluridine (5mU) (Extended Data Fig. 3h), which support translation while variably decreasing innate immune response^42^. Template RNAs could be fully uridine-substituted as well, with %GFP^+^ cells and median GFP intensity being highest using template RNAs with ψ replacing uridine (Fig. 2h), independent of the choice of transgene promoter (Extended Data Fig. 3f). Droplet digital PCR (ddPCR) to quantify the copy number of transgene 3′ and 5′ ends revealed that ψ modification of template RNA increased both the total number of insertions monitored by transgene 3′ end detection and the percentage of insertions that were full-length (Fig. 2i and Extended Data Fig. 3g). About 50% full-length insertion was obtained, even without yet having knowledge of optimal template RNA base composition, structure formation or purification.

Under 2-RNA delivery conditions favorable for full-length insertions (ψ template RNA encoding a GFP transgene with CMV promoter, cotransfected with ZoAl mRNA), many human, monkey and mouse cell lines could acquire and express transgenes (Extended Data Fig. 3i–l). In particular RPE and ARPE-19 (human epithelial cell lines), as well as IMR-90 and MRC-5 (human fibroblast cell lines), had more GFP^+^ cells and up to around 20 times higher average GFP ORF copy number in the entire transfected cell pool than transformed human cell lines such as HEK293T and HeLa (Extended Data Fig. 3j,k), perhaps related to lower rDNA copy number in the transformed cell lines (Extended Data Fig. 3k, right side *y* axis) and/or increased rDNA array instability^23,43^. All cell lines gained the 3′ and 5′ junctions expected for precise rDNA insertion of full-length transgenes (Extended Data Fig. 3l).

We used the ARPE-19 retinal pigmented epithelium cell line to demonstrate the function of human TERT expressed from an rDNA-inserted transgene. TERT expression is typically limiting for telomerase activity^44^. Telomerase activation confers human somatic cells with greatly extended proliferative capacity that could rescue proliferative deficiencies in several human diseases^44^. ARPE-19 cells were subject to 2-RNA transfection using template RNA encoding CMV promoter, 3.4 kb TERT ORF and polyA signal (Fig. 2j, top). Transfection of template RNA with ZoAl mRNA, but not ZoAl-RTD mRNA, generated transgene insertions to rDNA detected by PCR of 3′ and 5′ junctions (Fig. 2k). Cell extracts produced 1 d post-transfection were assayed for primer extension with telomeric repeats using standard quantitative and gel-based telomerase activity assays. Cells transfected with ZoAl-RTD mRNA and template RNA gained little if any telomerase activity, whereas cells transfected with WT or endonuclease-adjusted (below) ZoAl protein had elevated telomerase activity (Fig. 2j and Extended Data Fig. 3m). This result demonstrates transgene insertion using a template RNA of 4.5 kb.

### Transgene expression stability

**Fig. 3:**
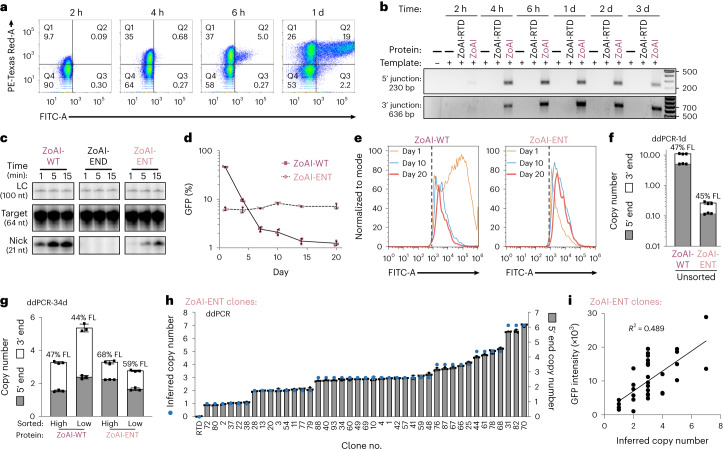
Transgene insertion efficiency and transgene expression stability. **a**,**b**, Time course of mCherry and GFP expression following 2-RNA transfection with mCherry mRNA and GFP transgene template RNA. RNA dose was 1 μg 2-RNA system with 0.03 μg mCherry mRNA. Transfection solution was replaced with fresh media 1 h post-transfection. **a**, Flow data for a representative replicate with *y* axis mCherry intensity and *x* axis GFP intensity. **b**, PCR detection of transgene junctions in a representative replicate. **c**, Denaturing PAGE resolution of TPRT reaction products was done on a single gel with different size ranges cropped. **d**,**e**, Flow results comparing transfected cell pools of ZoAl-WT and ZoAl-ENT for %GFP^+^ cells over continuous culture. RNA dose was 1.5 μg. The dashed line in **e** indicates the gating used to remove GFP-negative cells. **f**,**g**, ddPCR copy number calculations. **f**, Note the log-scale *y* axis. **g**, ddPCR was performed on GFP^+^ cells re-sorted at day 34. The white bar starts from the *x* axis, not from the top of the gray bar. **h**, Clonal GFP^+^ cell lines generated by ZoAl-ENT were assayed by ddPCR. Inferred copy number (blue dot) is an adjustment of ddPCR result to an integer, assuming slight under-replication of rDNA relative to reference genes in the asynchronous cell populations. **i**, Correlation of GFP intensity with full-length transgene copy number across ZoAl- ENT clonal cell lines. *R*^2^ represents the Pearson correlation coefficient from linear regression. In any relevant panel, data are presented as mean values ± error bars indicating standard deviation for three technical replicates.

**Extended Data Fig. 4:**
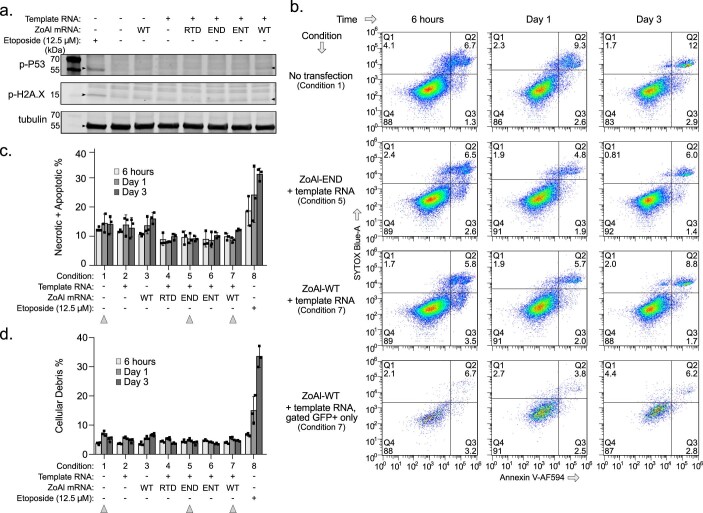
Survey of DNA damage response after 2-RNA delivery. **a**, PRINT triggering of DNA damage response was analyzed by immunoblot analysis of phosphorylated P53 (serine 15), $\gamma$-H2A.X, and tubulin (loading control) 6 hours after 2-RNA transfection of 6 μg RNA per 10 cm dish. Cell treatment with 12.5 μM etoposide was a positive control. Tubulin and phosphorylated P53 were probed on the same gel, $\gamma$-H2A.X was processed in parallel loading the same amount of sample on a separate gel. **b**, Flow data for a representative replicate of cell toxicity assays, using conditions indicated with a gray triangle in **c-d**. SYTOX Blue-A staining on the y-axis indicates loss of membrane integrity and mCherry-A values on the x-axis are Annexin V staining indicative of apoptotic cells. The final row shows only GFP+ cells from the data immediately above. RNA dose was 1 μg. **c**, Bar graph of % cells positive for Annexin V or SYTOX staining used the sum of the upper quadrants and the lower right quadrant from flow plots exemplified in **b**. **d**, Bar graph of % of cell detections gated as debris. In any relevant panel, data are presented as mean values +/- error bars indicating standard deviation for 3 technical replicates.

We investigated the kinetics of GFP transgene insertion and expression in RPE cells transfected with the 2-RNA system with spiked-in mCherry mRNA as a transfection reporter. Translation of the mCherry mRNA gave fluorescent mCherry protein detectable starting at 2 h and in 35% of cells by 4 h post-transfection (Fig. 3a). Transgene–rDNA junctions were weakly detectable at 2 h and approached maximum detection by 4 h (Fig. 3b). GFP fluorescence was readily detectable by 6 h and higher at the following 1 d time point (Fig. 3a). Of note, very little if any DNA damage response induction was detected on 2-RNA delivery, as monitored by phosphorylation of p53 serine 15 or phosphorylation of histone H2A.X (Extended Data Fig. 4a). There was little if any increase in cells positive by Annexin V and/or SYTOX staining, which detect apoptotic and necrotic cells, measured at 6 h, 1 d or 3 d post-transfection (Extended Data Fig. 4b–d).

**Extended Data Fig. 5:**
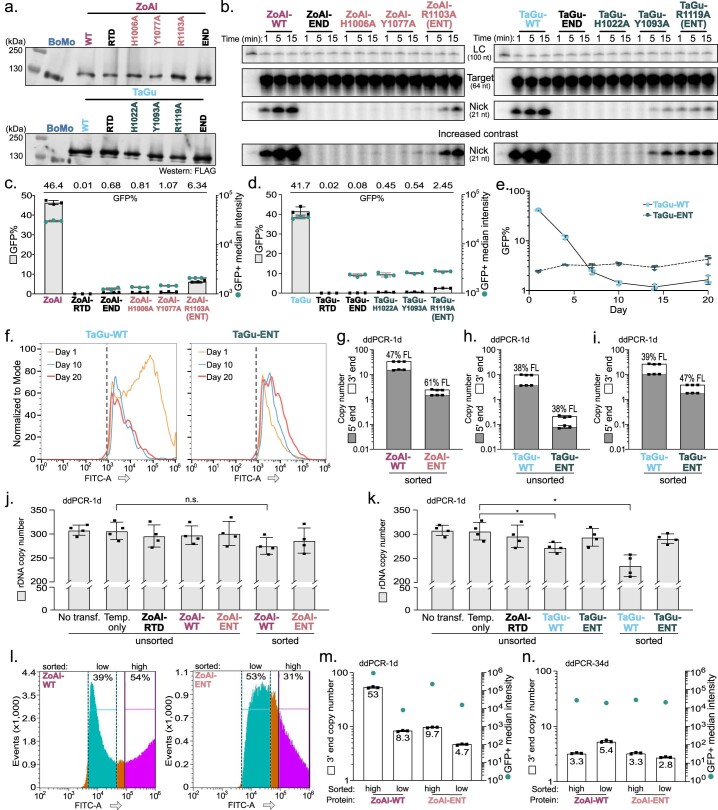
Biochemical and biological comparison of R2 proteins varying in EN activity. **a**, Immunoblot analysis of affinity purified R2 proteins. **b**, Denaturing PAGE resolution of reaction products for the time indicated in minutes was done on a single gel each for ZoAl and TaGu variants. Different size ranges were cropped from full-gel imaging. To detect cleavage without subsequent TPRT, template RNA was GeFo 3’UTR lacking an R4 3’ tail and no dNTPs were added to the reaction. An enhanced contrast image of the nicked product is included to detect low activity levels. **c-d**, Flow results for transgene insertion using WT or mutants of ZoAl (**c**) or TaGu (**d**). RNA dose was 1.5 μg. **e**, Transgene-encoded GFP was monitored over continued passage of unsorted cell pools from 2-RNA delivery using TaGu-WT or TaGu-ENT. **f**, Histogram of GFP intensity in GFP+ cells from representative samples in **e**. Dashed line indicates the gating used to remove GFP-negative cells. **g-i**, ddPCR results for unsorted or sorted cell pools assayed 1 day post-transfection. Note that the white bar starts from the x-axis, and the log scale for y-axes. **j-k**, ddPCR determination of total rDNA copy number 1 day post-transfection. Mann-Whitney U tests were used to compare to the template-only transfected cells as the normalization standard. Significant p-values of <0.05 are indicated with *. **l**, One day following 2-RNA delivery, cells were sorted into higher (magenta) and lower (teal) GFP intensity pools. Note that the relative percentage of cells in each pool differs for transgene insertion by ZoAl-WT or ZoAl-ENT. **m-n**, ddPCR was used to determine average number of insertions to rDNA for the cell pools in **l** and for re-sorted GFP+ cells in these cell pools after 34 days of continuous growth. Right y-axis indicates median GFP intensity of GFP+ cells. Note the log-scale y-axes. In any relevant panel, data are presented as mean values +/- error bars indicating standard deviation for 3 technical replicates.

With continuous passaging of the pool of transfected cells, %GFP^+^ decreased with kinetics suggesting that the cells contributing to population expansion were mostly GFP-negative. To test the influence of transgene copy number on outgrowth of GFP^+^ cells, we designed and purified ZoAl and TaGu sequence variants intended to diminish but not eliminate EN activity (Extended Data Fig. 5a,b), guided by studies of bacterially expressed BoMo^45^. Biochemical assays of target-site cleavage and TPRT showed that ZoAl-R1103A had tuned-down EN activity that was still precisely positioned at the target site (Fig. 3c and Extended Data Fig. 5b). Parallel results were observed for the corresponding TaGu-R1119A (Extended Data Fig. 5b). We describe these variants as ‘EN-tuned’ (ENT) to distinguish them from EN active-site mutations that eliminate detectable nicking activity (END; Fig. 3c and Extended Data Figs. 1c,d and 5b). In RPE cells assayed 1 d after 2-RNA delivery, ZoAl-ENT gave roughly 6% GFP^+^ cells, reduced from roughly 45% using WT ZoAl (Fig. 3d and Extended Data Fig. 5c). TaGu-ENT gave fewer GFP^+^ cells, only roughly 2%, reduced from roughly 40% for the WT protein (Extended Data Fig. 5d,e). Both ENT proteins generated transgenes with the expected rDNA junctions, as detected by PCR and genome sequencing (below). In a striking manner, use of an ENT protein eliminated the decline in %GFP^+^ cells with culture outgrowth (Fig. 3d and Extended Data Fig. 5e). The pool of GFP^+^ cells generated by ZoAl-ENT or TaGu-ENT showed an initial increase of GFP intensity and subsequent stable maintenance, in contrast to the reduction of GFP intensity that occurs with proliferation after transgene delivery using the WT proteins (Fig. 3e and Extended Data Fig. 5f).

We suspected that the GFP expression stability obtained using ENT proteins derived from reduced transgene copy number per cell. To compare inserted transgene copy numbers, we used ddPCR to quantify the total number of insertions interrupting rDNA units (assaying for the transgene 3′ end) and the number of full-length transgenes (assaying for the transgene 5′ end). The unsorted pool of 2-RNA transfected cells with ZoAl-ENT had an average of 0.2–0.3 total insertions per cell, compared to the ZoAl-WT average of roughly ten total insertions per cell (Fig. 3f). Therefore, ZoAl-ENT decreased insertion copy number roughly 40-fold, without changing the roughly 50% ratio of full-length to total insertions. We next repeated ddPCR quantifications using sorted GFP^+^ cells, which showed a similar differential for insertion copy number (ZoAl-WT average of 33 and ZoAl-ENT average of 2.5 per cell) and an enrichment for full-length transgenes in ZoAl-ENT cells (Extended Data Fig. 5g). Parallel results were observed in comparisons of TaGu-WT and TaGu-ENT proteins, which compared to the ZoAl proteins had a slightly lower percentage of full-length transgene insertions (Extended Data Fig. 5h,i). Maximal transgene copy number correlated with a reduction in rDNA copy number that was notable for TaGu-WT (Extended Data Fig. 5j,k).

We sorted GFP^+^ cells generated with ZoAl-WT and ZoAl-ENT into higher versus lower GFP intensity pools at day 1 after 2-RNA delivery (Extended Data Fig. 5l), with average transgene 3′ end copy number ranging from 53 to 4.7 (Extended Data Fig. 5m). We then passaged each cell pool in continuous growth and sorted again for GFP^+^ cells at day 34 after 2-RNA delivery. In 1 month of proliferation, average insertion copy number in GFP^+^ cells declined greatly in ZoAl-WT high-intensity GFP^+^ cells, from an average of 53 to 3.3 rDNA insertions per cell, and more modestly in the other three cell pools (Extended Data Fig. 5n). Median GFP intensity in the cell pools tracked with insertion copy number (Extended Data Fig. 5m,n). The total insertion copy number maintained stably with proliferation was in the range of 2.8 to 5.5 with 44 to 68% full-length transgenes (Fig. 3g).

**Extended Data Fig. 6:**
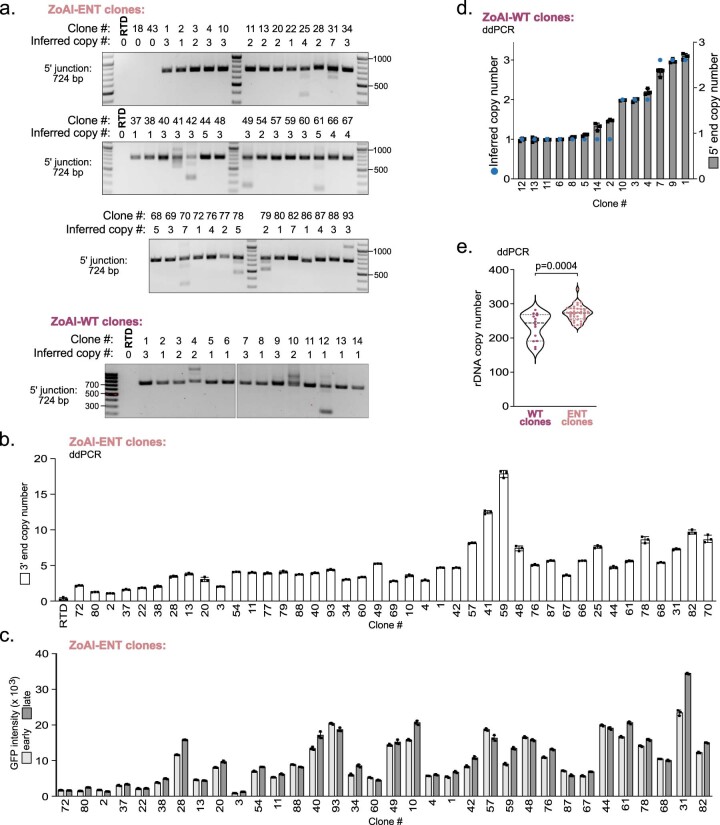
Additional clonal cell line analysis. GFP+ cells generated by ZoAl-ENT or ZoAl-WT were sorted into single cells and expanded to generate clonal cell lines. **a**, PCR detection of 5’ transgene junctions in gDNA from clonal cell lines. The weak PCR products were detected intermittently across re-cloned cell lines, suggestive of PCR artifacts. ZoAl-ENT clone 86 had a small 5’ truncation. **b**, ddPCR values for transgene 3’ end copy number for clonal cell lines with ddPCR values and inferred transgene 5’ end copy number shown in Fig. 3h. Clones are rank-ordered by increasing transgene 5’ copy number. **c**, Median GFP fluorescence intensity in cell lines from early or late time points (see Methods). This comparison used clonal cell lines with closely matched population doubling time at early and late time points to exclude an influence of different cell cycle timing. Clones are rank-ordered by increasing transgene 5’ copy number. **d**, ddPCR determination of full-length transgene copy number in GFP+ clonal cell lines generated with ZoAl-WT. Inferred transgene copy number (blue dot) is an adjustment of ddPCR results to an integer assuming slight under-replication of rDNA relative to reference genes in the asynchronous cell populations. **e**, Total rDNA copy number for clonal cell lines illustrated with violin plots marking the upper and lower quartiles alongside the median; n = 14 WT clones and 42 ENT clones. A two-sided Mann-Whitney U test was used to compare clones from the two populations. In any relevant panel, data are presented as mean values +/- error bars indicating standard deviation for 3 technical replicates.

To additionally confirm that rDNA insertions do not compromise cell growth if their copy number is limited to a low range, we generated clonal cell lines. We sorted single GFP^+^ cells at day 1 after 2-RNA delivery of ZoAl-WT or ZoAl-ENT with template RNA encoding GFP expression cassette and then let them proliferate clonally. Matching the cell pools, most GFP^+^ cells generated by ZoAl-WT did not generate GFP^+^ clonal cell lines, whereas most of the GFP^+^ cells generated by ZoAl-ENT remained GFP^+^. Transgene insertion to rDNA was confirmed in each clonal cell line by PCR (Extended Data Fig. 6a). Full-length transgene copy number in ZoAl-ENT clonal cell lines ranged from 1 to 7 (Fig. 3h) and transgene 3′ end copy number was generally lower than ten (Extended Data Fig. 6b). GFP^+^ clonal cell lines retained consistent GFP intensity over 2 months of continuous culture (Extended Data Fig. 6c), and GFP intensity was generally correlated to full-length transgene copy number (Fig. 3i). Some GFP^+^ cell lines had only a single insertion that was a full-length transgene. Among the few GFP^+^ clonal cell lines obtained using ZoAl-WT, full-length transgene copy number ranged from 1 to 3 (Extended Data Fig. 6d), mirroring the stable copy number when high-intensity GFP^+^ cells were cultured as a bulk cell pool (Extended Data Fig. 5n). Also consistent with the bulk cell pools, many ZoAl-WT but not ZoAl-ENT clonal cell lines had reduced rDNA copy number (Extended Data Fig. 6e), suggesting that initially high transgene copy number results in loss of transgene-containing rDNA units with proliferation.

### Insertion-site specificity

R2 retroelements in most species, including ZoAl and TaGu, are present in genomes only at the rDNA target site^12,46^. To confirm that transgene insertion has this site specificity in human cells, we performed Illumina whole-genome sequencing (WGS) on pooled GFP^+^ RPE cells following 2-RNA delivery. We compared ZoAl-WT and TaGu-WT insertions using CBh-promoter template RNA made with uridine and also compared ZoAl- and TaGu- WT versus ENT insertions using CMV-promoter template RNA made with ψ. Reads were first mapped to transgene sequence joined to 28 S rDNA with the precise 5′ and 3′ junctions generated by base pairing of introduced sequence to the target site: template R4 3′ tail annealing to downstream target site, and cDNA 3′ 28 nt annealing to upstream target site. Any nonaligned portion of a transgene-mapping read was aligned to a full-length rDNA unit to detect deletion or duplication flanking the target site. If not mapped to rDNA, the next mapping was to the human genome. In addition to examining reads containing the transgene sequence, we examined rDNA target-site sequence reads to detect any TPRT-mediated insertions other than the intended transgene sequence, but no such events were observed.

**Fig. 4:**
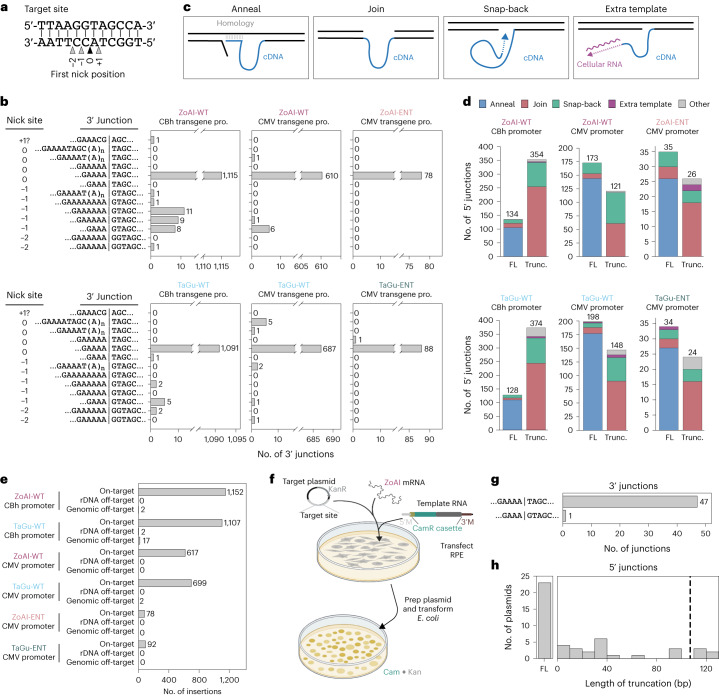
Site specificity of transgene insertion. **a**,**b**, Schematic (**a**) and tabulation (**b**) of inferred nick positions and sequenced transgene 3′ junctions. pro., promoter. **c**,**d**, Schematics (**c**) and tabulation (**d**) of transgene 5′ junction categories. Trunc., truncated. **e**, Tabulation of transgene insertion sites based on 3′ junction reads. **f**, Illustration of plasmid-based transgene insertion assays. Template RNA encoded a chloramphenicol resistance (CamR) cassette for bacterial selection of insertion-containing plasmids. The target-site plasmid backbone encoded kanamycin resistance (KanR). Created with BioRender.com. **g**,**h**, Analysis of transgene junctions determined by nanopore sequencing of 48 insertion-containing plasmids: 3′ (**g**) and 5′ (**h**). In **h**, dashed vertical line indicates the location of the CamR stop codon (note that the expression cassette is in reverse, rRNA-antisense orientation).

**Extended Data Fig. 7:**
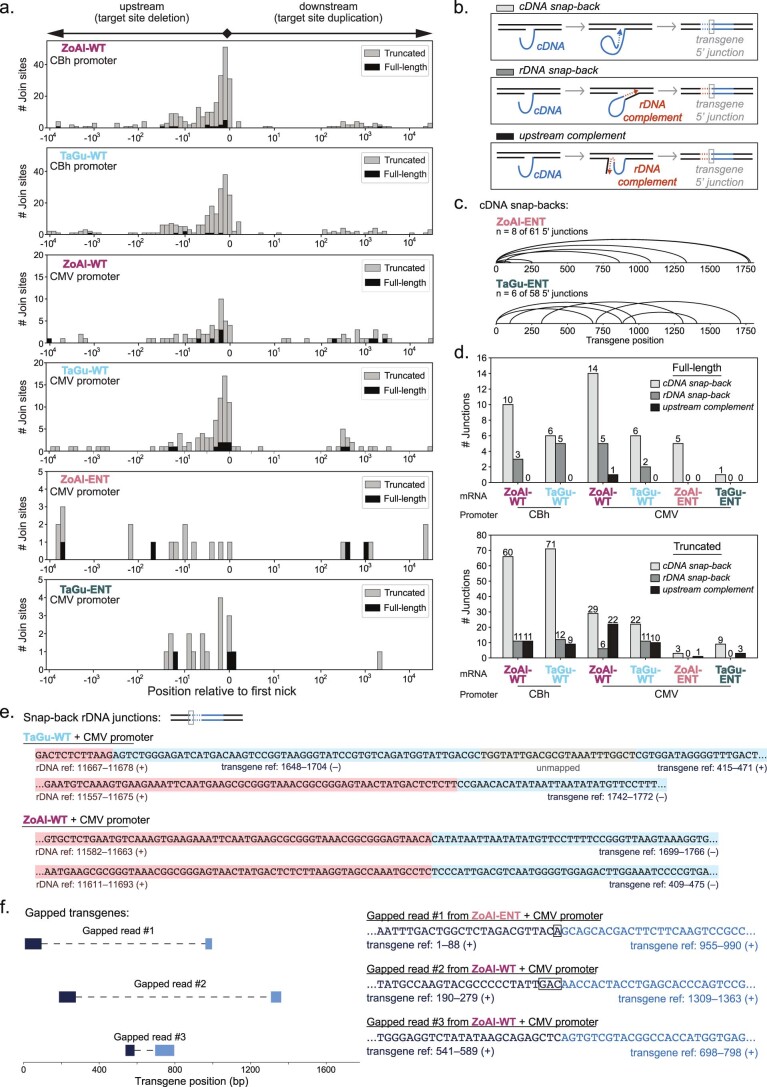
Analysis of on-target transgene insertions. **a**, rDNA positions of 5’ junction joining for full-length and 5’-truncated transgene insertions are plotted relative to the first-strand nick position. **b**, Junctions in the snap-back category are sub-classified by where a cDNA 3’ end putatively primed additional DNA-templated DNA synthesis. The 3’ end of cDNA synthesis from transfected template RNA is considered the transgene 5’ junction. Snap-back events can generate rDNA junction fusions to antisense transgene cDNA (see panel **e** for examples). **c**, Illustration of where a cDNA 3’ end (left side of arch) putatively primed additional synthesis on an internal position of cDNA (right side of arch). cDNA snap-back profiles were similar across variants of a protein and are shown for ZoAl-ENT and TaGu-ENT as representative. **d**, Counts for sub-categories of snap-back junction. **e**, Examples of rDNA junctions to antisense transgene sequence, indicative of cDNA snap-back synthesis. **f**, Examples of putative internally gapped transgene sequence reads. Some but not all gaps have potential microhomology between joined transgene sequences, indicated by a rectangular box. In **e**,**f** (+) indicates sequence expected for the rRNA-sense strand whereas (-) indicates sequence expected for the antisense strand.

Transgene 3′ end junctions were almost entirely a seamless join of R2 3′ UTR to the target site, guided by template 3′ tail annealing to nicked target site (Fig. 4a,b, position 0). Infrequently, on the order of 1% of insertions for ZoAl and more rarely for TaGu, an extra guanosine was present at the junction, suggestive of occasional nicking 1 bp upstream of the canonical position (Fig. 4a,b, position −1). At transgene 5′ ends, full-length transgene junctions were predominantly a seamless join from annealing of the first-strand cDNA 3′ end and upstream target site rDNA (Fig. 4c, anneal and Fig. 4d, blue bars). A fraction of full-length transgene insertions did not have this precise 5′ junction: for example, instead having a direct join that duplicated part or all of the 28 nt present in both template and target site (Fig. 4c, join and Fig. 4d, red bars). Join was the predominant 5′ junction category of 5′-truncated transgenes (Fig. 4d, red bars). On the rDNA side of the 5′ junction, almost all insertions fused the transgene to rDNA within 100 bp of the nick site, and most were immediately adjacent (Extended Data Fig. 7a). In a third category of junctions designated snap-back^47^^,48^ (Fig. 4c), before 5′ junction formation, the cDNA 3′ end appeared to prime additional DNA synthesis complementary to the cDNA or less frequently nearby rDNA (Fig. 4d, green bars and Extended Data Fig. 7b–d). For consistency, we considered the RNA-templated cDNA 3′ end to be the bona fide transgene 5′ end (Extended Data Fig. 7b). We did detect upstream rDNA junctions to the antisense-orientation transgene sequence, as expected for cDNA snap-back synthesis before rDNA 5′ junction formation (Extended Data Fig. 7e). Especially for ZoAl, snap-backs occurred after synthesis of full-length cDNA (Extended Data Fig. 7c). As a fourth category of transgene 5′ junction, we detected transgene sequence fusions to a noncoding RNA sequence, almost always U6 RNA (Fig. 4c, extra template, Fig. 4d, purple bars and sequences in Supplementary Table 1c). These junctions could result from template jumping and/or ligation of the template RNA 5′ end to a noncoding RNA before cDNA synthesis^49^. Some transgene sequence reads were suggestive of internal transgene deletions, but their context as snap-back structures or repair products or TPRT-synthesized cDNA is not resolved (examples in Extended Data Fig. 7f).

**Extended Data Fig. 8:**
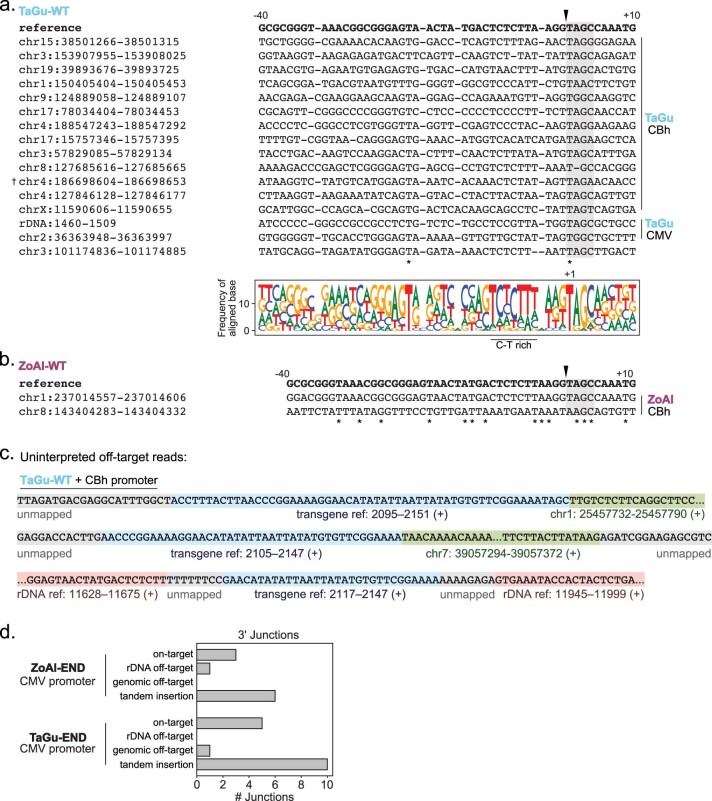
Analysis of off-target transgene sequence reads. **a-b**, Sequence alignment of the reference rDNA target site and genomic loci of candidate off-target TPRT by TaGu (**a**) and ZoAl (**b**). Each sequence is 40 bp upstream + 10 bp downstream of the inferred nick position on the non-primer strand, with black arrowhead indicating the inferred nick position. An asterisk indicates identical base identity for all sequences at the aligned position. Gray shading indicates the target site region anticipated to base-pair with the template RNA 3’ tail. The dagger in (**a**) indicates an insertion sequenced more than once. Below TaGu off-target insertions, a sequence logo is included. **c**, Read sequences from the off-target category that lack a TPRT signature. The (+) indicates sequence expected for the rRNA-sense strand. **d**, Distribution of 3’ transgene junction read counts for ZoAl-END and TaGu- END.

**Extended Data Fig. 9:**
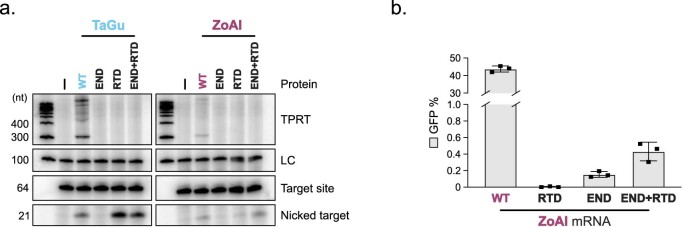
Coordination of target-site nicking and TPRT. **a**, In vitro TPRT activity assay for TaGu and ZoAl variants WT, END, RTD, or mixed END and RTD. Different size ranges were cropped from imaging after denaturing PAGE. **b**, PRINT was performed using ZoAl variants WT, END, RTD, or mixed END and RTD. In both assays, the RTD + END mixture had twice the amount of total protein or mRNA. Data is presented as mean values +/- error bars indicating standard deviation for 3 technical replicates.

To assess potential off-target transgene insertions, we first analyzed the few transgene 3′ junction reads that deviated by more than 2 bp from a precise fusion to the R2 rDNA target site. Rare reads that fused an internal region of transgene sequence to a genomic location would arise from snap-back synthesis (Extended Data Fig. 7e) or other non-TPRT event, because TPRT requires the template 3′ module. On the other hand, transgene 3′ junctions that include the template 3′ end are candidate off-target insertions. These were detected at a higher frequency for TaGu than ZoAl (roughly 1% versus 0.1%; Fig. 4e, off-target; sequences in Supplementary Table 1d). Putative off-target genomic insertion sites shared little sequence identity with the rDNA target site, other than having a primer-complementary sequence immediately downstream of the inferred first-strand nick (Extended Data Fig. 8a,b). Three TaGu-WT 3′ junction reads with very short transgene length and nonmapping sequence are of uninterpreted origin (Extended Data Fig. 8c). We also analyzed transgene synthesis by END proteins, to investigate whether lack of EN activity enhanced off-target insertions. In WGS from scarce sorted GFP^+^ cells, the major category of transgene 3′ junctions was head-to-tail tandem repeats (Extended Data Fig. 8d and sequences in Supplementary Table 1e), possibly generated by tandem template copying. The rarity of ZoAl-END or TaGu-END transgene synthesis suggests that TPRT is dependent on R2 protein-mediated nicking of a target site, with single-stranded primer hand-off from the EN active site to the RT active site^16^. Consistent with a concerted hand-off, combining RTD and END proteins did not give efficient TPRT in vitro or PRINT in cells (Extended Data Fig. 9).

To additionally confirm full-length transgene synthesis, we performed nanopore consensus sequencing of insertions to the rDNA target site introduced on a plasmid (Methods). We cotransfected plasmid containing the rDNA target site with ZoAl mRNA and template RNA encoding a transgene for bacterial antibiotic resistance (Fig. 4f, top). After recovering plasmids from transfected RPE cells, they were transformed to *Escherichia coli* and colonies were selected for the combination of kanamycin and chloramphenicol resistance, conferred by the plasmid backbone and transgene, respectively (Fig. 4f, bottom). Mirroring insertions to rDNA loci in the genome, the fidelity of 3′ junction formation was high (Fig. 4g) and both full-length and 5′-truncated transgenes were recovered (Fig. 4h). We estimated the combined error rate of T7 RNAP synthesis of template RNA in vitro and ZoAl synthesis of cDNA in cells by using nanopore consensus sequences of the roughly 300 bp template 3′ module, which is under minimal purifying selection compared to the transgene payload. We detected only one error per roughly 10,000 bp, consistent with the expected fidelity of RNA and cDNA synthesis^50,51^. Overall, results above constitute proof-of-principle for a 2-RNA delivery approach to supplement the human genome with transgenes of choice, including autonomous cassettes for production of therapeutic proteins and/or RNAs.

## Discussion

This work conclusively demonstrates site-specific insertion of full-length transgenes by the 2-RNA system of PRINT. The TPRT step is a native non-LTR retroelement mechanism that supports successful retroelement propagation in eukaryotic genomes^52^. PRINT exploits native R2 protein finesse of coordinated, codependent target-site binding, nicking and primer hand-off for TPRT. Concerted nicking and cDNA synthesis may avoid additive off-target activities from linking together autonomous modules for DNA binding, nicking and/or DNA synthesis^53,54^. PRINT also relies on a nonnative separation of protein-encoding mRNA from template RNA. Additional effort is necessary to understand and optimize the specificity, efficiency and stability of R2 protein interaction with a separately provided template RNA in cells.

If PRINT is eventually developed into a gene therapy approach, it will complement rather than replace CRISPR–Cas-based methods of gene disruption, base editing and sequence replacement that all use a guide RNA to bring DNA synthesis and repair machinery to an endogenous gene locus^1^. Comparing methods for autonomous transgene delivery, PRINT is distinguished from many by a lack of reliance on donor DNA. Programmable site-specific nucleases rely on donor DNA to template gene-sized insertions^55,56^. Donor DNA can also be maintained as an episome^2^ or codelivered with recombinase enzymes for integration^57–59^. In any of these approaches, donor DNA must evade recognition by the innate immune response^6^, and its presence increases the risk of oncogenic genome mutagenesis^60^. The use of RNA instead of DNA to template DNA insertion occurs in endogenous DNA break repair, for example at loci with nascent transcripts in high local proximity to the break site^40,41^. Prime editing brings template RNA to a genome locus by its fusion to the guide RNA of a Cas-protein nuclease, followed by retroviral RT extension of an annealed DNA–RNA substrate^54^. Prime editing, with or without codelivery of a site-specific integrase and donor DNA^54,61^, and retron-based technologies, which use a bacterial RT to make extragenomic cDNA local to the site of intended DNA repair^62,63^, differ from PRINT in their limits on insertion length and more importantly on their likely off-target and/or cytoplasmic RT activities. PRINT also differs from mRNA delivery^64,65^ in resistance to dilution by cell division and mRNA decay.

Using rDNA as a target site meets the criteria for safe-harbor insertion, with minimal risk of disruptive or oncogenic impact on flanking chromosome regions^66,67^. Consistent with this expectation, transgenes have been stably expressed from rDNA in pluripotent cells and animals^24–33^. Furthermore, in human cells, the rDNA-dedicated five acrocentric chromosome arms segregate into nucleolar compartments with highly privileged DNA repair: DNA breaks are repaired preferentially by nonhomologous end joining, but as a back-up rDNA breaks are translocated to the nucleolar periphery and repaired by homologous recombination in all stages of the cell cycle^68,69^. Epigenetic silencing of approximately half of the rDNA occurs after embryogenesis, and only those silenced repeats have rRNA-coding regions packaged into nucleosomes^18^. Despite the advantages of rDNA as a safe-harbor locus, for some applications it could be advantageous to retarget. Because the R2 family includes retroelements with relaxed or even redirected target-site specificity^12,14,16^, there is potential for retargeting without corrupting the allosteric coordination of RNA binding, target-site binding, first-strand nicking and TPRT.

Critical knowledge gaps and directions for PRINT development remain to be addressed. First, the cellular processes that govern pathway choice for transgene 5′ junction formation and second-strand synthesis are not defined. Knowledge of the DNA intermediates created during transgene synthesis will be important for assessing the risk of genome instability and strategies for suppression of transgene truncations. Second, this work does not address the relationship between transgene length and insertion efficiency. We suggest that exploring this question will benefit from developing a method to enrich template RNA molecules harboring intact 5′ and 3′ ends, to reduce the confounding influences of length-dependent increase in incomplete and fragmented RNA transcripts. Third, it will be essential to monitor and minimize undesirable cellular responses to introduced RNA. PRINT template RNA and mRNA could be additionally engineered with this goal in mind. Fourth, long-term transgene persistence should be investigated in contexts relevant to disease therapy. Fifth, PRINT efficiency across cell types, including nondividing cells, is of interest to investigate and understand. Transgene insertion using donor DNA to template repair of an induced DNA break is restricted in the cell cycle and in nondividing cells, due to suppression of homologous recombination, so PRINT could have particular therapeutic utility in those cell types. Finally, PRINT is likely to benefit from additional protein engineering, since native retroelement proteins may have evolutionarily subdued activity to limit their imposition on the host genome. Fully harnessing the potential of eukaryotic non-LTR retroelement proteins for genome engineering applications will benefit from better understanding of the structural and biochemical principles for EN-domain activity and specificity.

## Methods

### Sequences

Construct sequences used in this work are provided in Supplementary Table 1a. Constructs for producing R2 proteins and GFP transgene template RNA will be available from Addgene. Codon-optimized ORFs and other DNA modules were purchased from GenScript. The ZoAl-RTD mutation is DD644-645AA, EN-dead double mutation is D1041A and D1054A, and EN-low mutations are H1006A, Y1077A and R1103A numbered from a chosen start site for synthetic ZoAl ORF. The TaGu-RTD mutation is DD660-661AA, EN-dead double mutation is D1057A and D1070A, and EN-low mutations are H1022A, Y1093A and R1119A numbered from a chosen start site for TaGu ORF. PCR product sequences used for transcription of short template RNAs are listed in Supplementary Table 1a. Oligonucleotide sequences are listed in Supplementary Table 1b. Design of the minimal polyA signal (minPA) used insights from previous work^70^. The CMV immediate–early promoter has a single base-pair substitution intended to reduce transcriptional silencing by DNA methylation, based on previous work^71^. The SV40 immediate–early promoter was also modified, including a more optimal TATA box designed using insights from previous work^72^.

### Cell culture

RPE and ARPE-19 cells were grown in DMEM/F12 (Gibco) supplemented with 10% fetal bovine serum (FBS) (Seradigm) and 100 μg ml^−1^ Primocin (InvivoGen). HEK293T, HeLa, IMR-90, MRC-5 and C2C12 cells were grown in DMEM (Gibco) supplemented with 10% FBS. Vero cells were cultivated in DMEM supplemented with 10% FBS and 1% nonessential amino acid (Gibco). All cells were cultured at 37 °C under 5% CO_2_ and tested for mycoplasma contamination. Human cell lines were validated by short tandem repeat profiling (Promega, catalog no. B9510).

### Protein expression and purification for biochemical assays

HEK293T cells were transiently transfected with pcDNA3.1 plasmids encoding proteins N-terminally tagged with a single FLAG peptide, unless stated otherwise. Cells at 80% confluency in a 10 cm plate were reverse transfected with 12 μg DNA using Lipofectamine 3000. After 16–24 h, cells were trypsinized, resuspended in 5 ml of media and pelleted at roughly 2,000*g* for 3 min in 15 ml conical tubes. Pelleted cells were washed by resuspension in 0.5 ml of chilled PBS containing 1 mM phenylmethylsulfonyl fluoride (PMSF), transferred to a 1.5 ml tube and repelleted at roughly 2,000*g* for 1 min at 4 °C. Cell pellets were resuspended in 4× pellet volume of 1× HLB (20 mM HEPES pH 8.0, 2 mM MgCl_2_, 200 μM EGTA, 10% glycerol, 1 mM DTT, 0.2% protease inhibitor cocktail (Sigma, catalog no. P8340), 1 mM PMSF) and set on ice for 5 min. Cells were then lysed by three cycles of snap freezing in liquid nitrogen and thawing in a room temperature water bath. Samples were then brought to 400 mM NaCl, gently vortexed and placed on ice for an additional 5 min. Lysed cells were spun at 17,000*g* for 5 min at 4 °C. The supernatant was collected and the concentration of NaCl lowered to 200 mM and NP-40 added by the addition of an equal volume of 1× HLB containing 0.2% NP-40. Samples were vortexed gently and spun at 17,000*g* for 10 min at 4 °C to clarify the supernatant.

For affinity purification, 20 μl of FLAG resin per sample (Sigma, catalog no. A2220) was equilibrated and blocked with 1 μg μl^−1^ molecular grade BSA and 1 μg μl^−1^ yeast tRNA (Calbiochem, catalog no. 55714) in 200 μl of immunoprecipitation buffer (1× HLB, 200 mM NaCl, 0.1% NP-40) for 30 min at 4 °C. Blocked resin was washed 2× with immunoprecipitation buffer and resuspended in 100 μl of immunoprecipitation buffer per sample, which was added to 700 μl of clarified cell lysate. Binding reactions were rotated at 4 °C for 2 h and then washed with immunoprecipitation buffer four times (two quick washes, two with 5 min of rotation at 4 °C). All buffer was removed with a 30G needle before bound resin was resuspended in 40 μl of immunoprecipitation buffer with 50 ng μl^−1^ 3× FLAG peptide (Sigma, catalog no. F4799) and incubated at room temperature for 1 h. The slurry was aliquoted, snap frozen and stored at −80 °C. Immunoblots used 0.45 μm nitrocellulose membrane (Bio-Rad, catalog no. 1620115) blocked in TBST (10 mM Tris-Cl pH 7.5, 150 mM NaCl, 0.1% Tween 20, 0.02% sodium azide) with 5% BSA and probed in the same buffer with anti-FLAG antibody (Sigma, catalog no. F1804, 1:3,000) and then Alexa Fluor 680 antimouse secondary (Thermo Fisher, catalog no. A21057, 1:2,000). Detection was by LI-COR Odyssey. Coomassie staining of affinity-purified proteins resolved by SDS–PAGE used recombinant MBP-BoMoC as a protein standard^73^.

### IVT

Template RNAs were transcribed with the HiScribe T7 Kit (NEB, catalog no. E2040S) according to the manufacturer’s instructions. RNA templates for biochemical assays of TPRT and for A-tract length change were made using 1 μg of PCR-amplified transcription template per 20 μl of reaction. Transgene template RNAs and mRNAs for cellular transfection were made using 1 μg per 20 μl plasmid fully linearized with Bbs I (NEB) for 4 h at 37 °C and then purified with PCR purification kit (QIAGEN, catalog no. 28106). Templates with TiGu 3′ UTR were instead digested using Sap I (NEB), due to an internal Bbs I site, and gel-purified with QIAEX II Gel Extraction Kit (QIAGEN, catalog no. 20021). R2 protein mRNAs were made with AG Clean cap (TriLink, catalog no. N-7113) per the manufacturer’s protocol^74^ using UTR sequences and DNA-templated, linker (L)-containing poly-adenosine tail A_30_L_10_A_70_ from the BioNTech COVID-19 vaccine mRNA^75^. Canonical ribonucleotides were purchased from NEB and uridine analogs were purchased from from TriLink or APExBIO. Transcription reactions were incubated at 37 °C for 2 h, followed by addition of 1 μl of DNase RQ1 (Promega, catalog no. M610A) or 2 μl RNase-free DNase I (Thermo Fisher, catalog no. FEREN0521). Product RNA was purified by desalting with a quick-spin column (Roche, catalog no. 11814397001) or illustra ProbeQuant G-50 Micro Column (Cytiva, catalog no. 28903408) followed by phenol–chloroform–isoamyl alcohol (PCI; Thermo Fisher, catalog no. BP1752I-100) purification and precipitation with final concentration of 2.5 M LiCl or with final concentration 0.3 M sodium acetate (pH 5.2) and 3 volumes of 100% ethanol. After washing with 70% ethanol 2–3 times, RNAs were resuspended in 1 mM sodium citrate (pH 6.5) or in water for RNAs used only for biochemical assays. Concentration was determined by NanoDrop and integrity verified by denaturing urea-PAGE with direct staining using SYBR Gold (Thermo Fisher, catalog no. S11494).

### TPRT assays with affinity-purified protein

The primer strand of target-site duplex was 5′ radiolabeled with ^32^P γ-ATP using T4 PNK (NEB, catalog no. M0201L). Unlabeled nucleotides were removed by spin column (Roche, catalog no. 11814397001 or Cytiva, catalog no. 27-5325-01). Complementary strands were annealed by heating to 95 °C and cooling by 1 °C per min. Unless indicated otherwise, TPRT template RNA was GeFo 3′ UTR_R4A22 with unmodified uridine. TPRT reactions were assembled in 20 μl with final concentrations of 25 mM Tris-HCl pH 7.5, 75 mM KCl, 5 mM MgCl_2_, 10 mM DTT, 2% PEG-6K, 5 nM target-site duplex, 0.6 μM template RNA, 0.5 mM dNTPs and approximately 10 nM R2 protein in immunoprecipitation elution buffer and then incubated at 37 °C for 30 min before heat inactivation at 70 °C for 5 min and dilution with 80 μl of stop solution (50 mM Tris-HCl pH 7.5, 20 mM EDTA, 0.2% SDS) spiked with 5′ radiolabeled 100-nt loading control oligonucleotide. Nucleic acid was purified by PCI extraction and ethanol precipitated in a dry ice ethanol bath. Samples were then pelleted at roughly 18,000*g* for 20 min at room temperature and pellets washed with 70% ethanol, resuspended in 5 μl of water and supplemented with 5 μl of formamide loading dye (95% deionized formamide, 0.025% w/v bromophenol blue, 0.025% w/v xylene cyanol, 5 mM EDTA pH 8.0). The sample was heated to 95 °C for 3 min and then placed on ice before loading half of the sample on a 9% urea-PAGE gel. After electrophoresis the gel was dried, exposed to a phosphoimaging screen and imaged by Typhoon Trio (Cytiva).

### PRINT by delivery of protein-encoding plasmid and template RNA

HEK293T cells were plated at 2.5 million cells per well in six-well plates and reverse-transfected with 1 µg plasmid using Lipofectamine 3000 at 1/2 mass/volume ratio as per the manufacturer’s instructions. On the next day, cells were split at a 1/2 ratio, keeping half. On the subsequent day, each well was reverse-transfected with 2 µg template RNA using Lipofectamine MessengerMAX (Thermo Fisher, catalog no. LMRNA015) at 1/2 mass/volume ratio as per the manufacturer’s instructions. Cells were collected 1 d after RNA transfection and the cell pellets were stored at −80 °C after snap freezing in liquid nitrogen.

### PRINT by 2-RNA delivery

RPE cells in log-phase growth at 50% confluency were replated at 0.75–1 million cells per well in six-well plates. Cells were reverse-transfected with mRNA and template RNA using Lipofectamine MessengerMAX at 1/2 mass/volume ratio as per the manufacturer’s instructions. For some experiments, ~0.03 µg of 5moU mCherry mRNA (TriLink, catalog no. L-7203) per 1 µg total RNA mixture was used as a spike-in transfection control. Cells were collected 20–24 h (1 d) after transfection unless noted otherwise. The same transfection protocol was used for other cell lines except with different cell density per well of a six-well plate: ARPE-19, HeLa, IMR-90, MRC-5 and Vero cells were plated at 1 million per well; C2C12 was plated at 0.5 milion per well and HEK293T was plated at 2 million per well. Unless noted otherwise, 2.5 µg RNA was transfected per well of six-well plate and mRNA/template molar ratio was 1/3. For transfections followed by sorting to single cells or graded GFP intensity cell pools, RNA dose ranged from 1–1.5 µg with 1/2 or 1/3 MessengerMAX.

Sequences for mRNA and template RNA transcription are provided in Supplementary Table 1a. Unless noted otherwise, the mRNAs encoding R2 proteins had 100% uridine substitution with 1mψ or 5moU. Protein expression used the FLAG-tagged ORF unless noted otherwise, except ZoAl-RTD was untagged. The template RNA 5′ module was either a minimal ribozyme (TCARZ) or a slightly longer 5′ UTR region (TCA5). In Extended Data Fig. 2, template RNAs were unmodified uridine with TCA5, the indicated 3′ UTR, and R4A22. In Extended Data Fig. 3d, template RNAs were unmodified uridine with TCA5, GeFo 3′ UTR, R4 and the indicated A-tract. Other template RNA transcripts were unmodified uridine with hairpinleader_TCA5_CBh_ORF_SV40PA_GeFo3′ UTR_R4A22 (Fig. 2c–g and Extended Data Fig. 3a,b) or pseudouridine with rRNAleader_TCARZ_CMV_ORF_minPA_GeFo3′ UTR_R4A22 (Figs. 2h–k and 3; CMV-promoter transgene WSG; Extended Data Figs. 3c,f,g,i–n, 4–6 and 9b). In Extended Data Fig. 3e,h and CBh promoter transgene WSG, template RNA was hairpinleader_TCARZ_CBh_ORF_SV40PA_GeFo3′ UTR_R4A22. In Extended Data Fig. 3f,g, all template RNAs had the TCARZ 5′ module for consistency; the CBh promoter had SV40PA and the CMV and SV40 promoters had minPA.

### Flow cytometry and cell sorting

**Extended Data Fig. 10:**
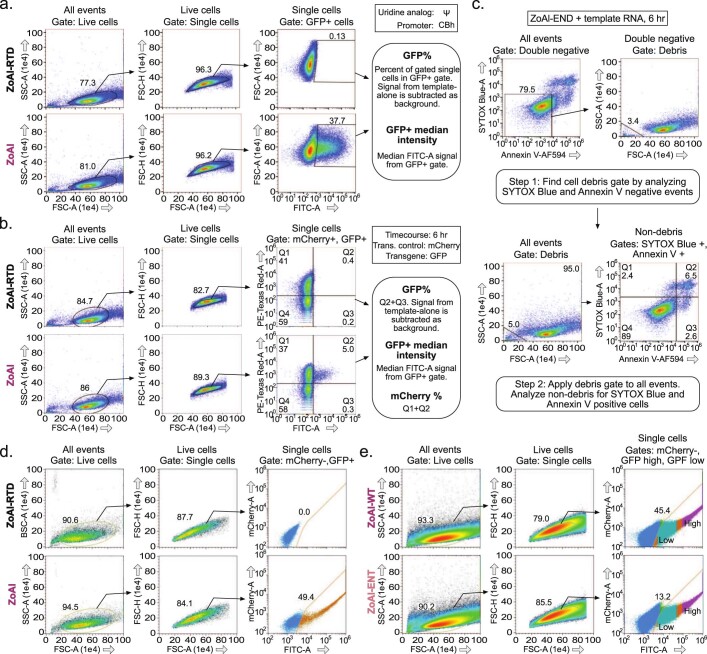
Flow cytometry gating strategies. **a**, Gating strategy used to analyze flow cytometry samples for GFP% and GFP+ median intensity. Representative images are from the promoter comparison test in Extended Data Fig. 3f. This strategy was used to analyze data displayed in Figs. 2c,d,f,h, 3d,e,i (y-axis), and Extended Data Fig. 3a,e,f,h, 5c–f,m,n (right y-axis), 6c, 9b. **b**, Gating strategy used to analyze flow cytometry samples co-transfected with mCherry mRNA as a transfection control. Representative images are from the time course in Fig. 3a. This strategy was used to analyze data displayed in Fig. 3a, and Extended Data Fig. 3c,i,j. **c**, Gating strategy used to analyze flow cytometry samples for DNA damage. Representative images are from Extended Data Fig. 4b. This strategy was used to analyze data displayed in Fig. 4b–d. **d**, Gating strategy for sorting GFP+ cells for outgrowth or sequencing. This sorting strategy was used for the generation of GFP+ pooled populations (Fig. 4b–e and Extended Data Fig. 5g,i–k, 7, 8) and GFP+ clones (Fig. 3h,i, and Extended Data Fig. 6). **e**, Gating strategy for sorting GFP+ high and low populations. The first steps are identical to (**d**) and differ in the creation of further gates (see Extended Data Fig. 5l) to delineate GFP+ high (magenta) and GFP+ low (teal) populations. This sorting strategy was used for the generation of the cell populations analyzed in Fig. 3g, and Extended Data Fig. 5l–n.

Cells were trypsinized to collect, and trypsin was inactivated by addition of the cell-appropriate medium with 5% FBS. Cell samples were analyzed by Attune NxT Flow Cytometer (Thermo Fisher) under the voltage setting of FSC 70V, SSC 280V, BL1 250V (for GFP) and YL2 250V (for mCherry). Cell sorting was done on Sony Sorter LE-SH800 equipped with 488 and 561 nm lasers using the 130 µm chip under ultra-purity sorting mode. Data analysis was performed in FlowJo (v.10.8.1). When gating for GFP^+^ or mCherry^+^ population, cells transfected with only template RNA or template RNA and ZoAl-RTD were used as negative controls. The %GFP^+^ was calculated by subtracting template-alone %GFP^+^ from the parallel 2-RNA transfection %GFP^+^. Median GFP intensity was determined using only the GFP^+^ cells in a population. For overlaid histograms of GFP intensity profiles, ‘Normalized to mode’ was used to scale the *y* axis for better cross-comparison. Error bars are from three technical replicates. Every assay had independent experimental replicates. Gating strategy for flow cytometry and cell sorting is visualized in Extended Data Fig. 10.

To make clonal cell lines, transfections used an RNA dose of 1–1.5 µg with 1/2 or 1/3 MessengerMAX. Single GFP^+^ cells were sorted to 96-well microtiter plates 1 d after transfection. Cells were allowed to proliferate for approximately 3 weeks before screening for GFP expression: 24% of expanded cell lines for ZoAl-WT were GFP^+^, whereas 94% of expanded cell lines for ZoAl-ENT were GFP^+^. At 6–7 weeks postsorting, cells were used for genotyping and ddPCR. For GFP intensity stability measurements, the time points were postsorting roughly 7 and 15 weeks of proliferation. Cells for the early time point were frozen at around 5 weeks and then returned to culture for 2 weeks before the 15 week time point to be able to measure GFP intensity on the same day.

### gDNA purification and junction PCR

Frozen cell pellets were thawed on ice and resuspended in 200 µl of RIPA lysis buffer (150 mM NaCl, 50 mM Tris-HCl pH 7.5, 1 mM EDTA, 1% Tx-100, 0.5% sodium deoxycholate, 0.1% SDS, 1 mM DTT). Each 200 µl of lysate was treated with 10 µl of 10 mg ml^−1^ RNase A (Thermo Fisher, catalog no. FEREN0531) at 37 °C for 30–60 min, followed by incubation with 5 µl of 20 mg ml^−1^ Proteinase K (Thermo Fisher, catalog no. FEREO0491) at 50 °C overnight. gDNA was then isolated by extraction with PCI and ethanol precipitation. After centrifugation, the aqueous layer was transferred to a fresh tube containing 50 µg glycogen, to which 1/10 volume 5 M NaCl and 3 vol 100% ethanol were added. gDNA was precipitated at −20 °C for at least 30 min. After a 30 min spin, gDNA pellets were washed 2–3 times with 75% ethanol, air-dried and resuspended in TE (10 mM Tris-HCl pH 8.0, 1 mM EDTA). gDNA prepared for WGS was instead dissolved in nuclease-free water. For PCR, 100–250 ng gDNA was used in a 25 µl of reaction with Q5 DNA polymerase (NEB). PCR primer sequences are listed in Supplementary Table 1b. PCR was as follows: 98 °C, 3 min (98 °C, 10 s; 65 °C, 30 s; 72 °C, 40 s per 1 kb) five times with annealing temperature decreasing by 1 °C per cycle (98 °C, 10 s; 60 °C, 30 s; 72 °C, 40 s per 1 kb) 25 times; 72 °C for 20 s. PCR products were analyzed on 1–2% agarose gels containing ethidium bromide and imaged using the Bio-Rad gel doc XR+ imaging system.

### Telomerase activity assays

One day after transfecting ARPE-19 cells with mRNA and RNA template, cells were collected for protein extraction. RNA dose was 1.5 μg with 6 μl of MessengerMax. Cell extract was prepared by hypotonic freeze–thaw lysis as described above, except with a final concentration of 150 mM NaCl. Quantitative telomeric repeat amplification protocol was performed using 2 µl of approximately 2 mg ml^−1^ cell extract by standard protocol^76^ with iTaq universal SYBR green Supermix (Bio-Rad) and a CFX96 Touch Real-Time PCR machine (Bio-Rad). Radiolabeled-nucleotide telomeric repeat amplification protocol assays were performed using 5 µl of 3-, 9- and 27-fold extract dilutions using standard protocol^77^ of primer extension followed by PCR, with imaging by Typhoon Trio (Cytiva).

### DNA damage assays

For relevant samples, drug treatment began 12 h before transfection. Medium was not changed and no additional drug was added at later time points. At indicated time points after 2-RNA delivery, cells were washed in PBS and trypsinized using minimal amounts of trypsin. Cells were resuspended in full-serum medium and allowed to recover for 20 min at 37 °C and 5% CO_2_. Cells were pelleted and washed in ice-cold PBS, and then resuspended in ice-cold Annexin binding buffer (10 mM HEPES pH 7.4, 140 mM NaCl, 2.5 mM CaCl_2_). A fraction of cells was subjected to Annexin V-AF594 (Invitrogen, catalog no. A13202) and SYTOX Blue (Thermo Fisher, catalog no. S34587) staining at room temperature for 15 min and then diluted for flow cytometry analysis. Collected data were analyzed according to R. Duggan’s method from the University of Chicago Flow Cytometry Core (https://voices.uchicago.edu/ucflow/2012/07/08/my-3-step-approach-to-gating-annexin-v-data-appropriately/). The double-negative fraction was gated for debris by very low forward and side scatter, well resolved from the live cell double-negative population.

For immunoblot analysis, 6 μg total of ZoAl mRNA and template RNA was transfected per 10 cm dish of RPE cells. At the indicated time points post-transfection, cells were washed and trypsinized and lysed as described above for protein purification. The supernatant was collected, and samples were normalized by total protein using Protein Assay Dye (Bio-Rad, catalog no. 5000006). Next, 60 μg of total protein was loaded in each lane of precast 4–15% TGX gels (Bio-Rad, 4561084). Protein was transferred to 0.2 μm nitrocellulose membrane (Bio-Rad, catalog no. 1620147), blocked in TBST (10 mM Tris-Cl pH 7.5, 150 mM NaCl, 0.1% Tween 20, 0.02% sodium azide) with 5% BSA and probed in the same buffer with rabbit anti-phospho-P53 (Ser15) (Invitrogen, catalog no. 14H61L24, 1:1,000), mouse anti-tubulin (Abcam, catalog no. ab44928, 1:1,000), or mouse anti-phospho-histone H2A.X (Ser139) (Invitrogen, catalog no. 6T2311, 1:1,000), followed by appropriate secondary, either Alexa Fluor 680 goat anti-rabbit (Invitrogen, catalog no. A21109, 1:2,000) or Alexa Fluor Plus 800 goat anti-mouse (Invitrogen, catalog no. A32730, 1:2,000). Detection was by LI-COR Odyssey. Because p-P53 and tubulin migrate similarly in SDS–PAGE, p-P53 was probed first and then tubulin.

### ddPCR

gDNA was digested overnight with Bam HI and Xmn I (NEB). Multiplex 24 µl ddPCR reactions were prepared by mixing 12 µl of ddPCR supermix (no dUTP; Bio-Rad, catalog no. 1863024), forward and reverse primers for target and reference genes (IDT, 833 nM final concentration each), probes complementary to target and reference amplicons (IDT; FAM for target and HEX for reference, 250 nM final concentration each) and digested gDNA at 1–5 ng µl^−1^ final concentration. Oligonucleotide sequences are listed in Supplementary Table 1b. Reaction mix was transferred to a DG8 cartridge (Bio-Rad, catalog no. 1864007) along with 70 µl of droplet generation oil (Bio-Rad, catalog no. 1863005), and droplets were generated in a Bio-Rad QX200 Droplet Generator. Following droplet generation, 40 µl was transferred into a 96-well plate and heat-sealed with pierceable foil. The droplets were thermal-cycled under the manufacturer’s recommended conditions with an annealing and/or extension temperature of 56 °C and analyzed using QX Manager software with default settings.

*RPP30* was used as the reference gene for all copy number analysis experiments. The copy number of RPP30 in each cell line was inferred using a panel of additional reference genes (*ALB*, *MRTFB* and *RPPH1*). We discovered that RPE and HeLa cells have an *RPP30* copy number per genome of three, whereas ARPE-19, 293T, IMR-90, MRC-5 and monkey Vero cells have an *RPP30* copy number of two. We were unable to determine *RPP30* copy number in mouse C2C12 cells, so quantification assumed a copy number of two per genome. Primers to detect *RPP30*, *ALB*, *MRTFB* and *RPPH1*, and rDNA were adapted from sequences previously described^78–82^. Inferred transgene copy number was adjusted to an integer assuming slight under-replication of rDNA relative to reference genes in the asynchronous cell populations.

### Genome sequencing and analysis

Cells were collected 1 d post-transfection. Purified gDNA was fragmented to 400–500 bp by Covaris shearing as part of Illumina library construction and NovaSeq 6000 PE150 sequencing performed by QB3 genomics facilities at UC Berkeley. Bioinformatic analyses were performed on the Berkeley Research Computing Savio cluster with SLURM job scheduling or on an Apple M1 Max processor. PCR and optical duplicates were removed with BBMap v.38.97 (https://sourceforge.net/projects/bbmap/) and reads were trimmed for quality with Trimmomatic v.0.39 (ref. ^83^). Reads shorter than 36 bp or with an overall PHRED quality less than 30 were discarded. All alignments were performed with bwa mem v.0.7.17 using default parameters^84^. Paired reads were aligned to transgene sequence precisely inserted between flanking 840 bp tracts of rDNA. Unmapped mates or portions of reads exceeding 20 bp were aligned to a complete rDNA unit using a consensus rDNA scaffold (GenBank KY962518.1). Read portions remaining unaligned were then mapped to the T2T-CHM13v2.0 human genome reference^85^. Finally, still-unaligned portions of reads too short for alignment by bwa mem were aligned to the rDNA reference or transgene template sequence with approximate string matching using fuzzysearch (https://github.com/taleinat/fuzzysearch). The following reads were then discarded: mate pairs without both reads mapped and spurious transgene-aligned reads (for example, reads aligning better to the human genome than to the transgene). To detect contaminating genetic material from pooled sequencing, reads were mapped to a curated list of observed contaminants, including the SARS-CoV-2 genome; reads mapping to these nonhuman sequences were discarded.

On-target reads were defined as those with transgene sequence and downstream rDNA beginning within 3 bp of the target-site nick. Off-target reads were defined as those with transgene sequence and (1) rDNA sequence not at the target site, or (2) downstream sequence mapping elsewhere in the human genome. Loci of putative off-target insertions were aligned to the reference target site with T-Coffee on the EMBL-EBI webserver^86^. The base frequencies at each position across aligned candidate TaGu off-target insertion sites were tallied and depicted with visualization tools from DeepLIFT^87^. To determine the initiation site of TPRT within on-target reads, fuzzysearch was used to find the 3′ end of transgene sequence (query sequence TGTTCGG on top strand after second-strand synthesis) and downstream rDNA sequence (query sequence TAGCCAA) within the read. The intervening sequence was used to infer nicking and initiation of TPRT.

Determination of the rDNA position of 5′ junction formation used the join category of junctions because anneal junctions are not informative. The 5′ junction category snap-back reads were identified by transgene-adjacent sequence mapping to the opposite strand of the transgene or rDNA scaffold. The 5′ junction category ‘other’ contained upstream sequences mapping somewhere in the genome other than rDNA joined to a transgene 5′ end. If sequence upstream of a 5′ transgene junction did not map, it was not classified. Only a strict subset of these reads were reclassified to the 5′ junction category ‘extra’ template, if by manual evaluation NCBI BLAST revealed that (1) the sequence mapped unambiguously to a single transcript or class of transcripts, (2) the insertion had correct strandedness for reverse transcribing an RNA and (3) reverse transcription began near or at the 3′ end of the annotated RNA transcript. The 5′ junction category tandem insertion reads was defined by the presence of upstream sequence mapping to the 3′ end of a transgene and downstream sequence mapping to the 5′ end of a full-length or truncated template cDNA. Any 5′ junction transgene reads without mapping portion were excluded. Internally gapped transgenes were identified by reads with upstream and downstream portions mapping noncontiguously to the same strand of the transgene reference. Microhomology at the junction was identified by comparing whether the last base on the upstream-aligned portion of the read matched the base on the reference sequence immediately before the downstream-aligned portion of the read. This procedure was repeated iteratively until the first nonmatching base was found. The same procedure was repeated for the other side of the junction, beginning with first downstream-aligned base and the base on the reference sequence immediately after the upstream-aligned portion of the read. The sum of these two iterative matching procedures was considered maximum possible microhomology.

### Plasmid insertion assays

Target plasmid backbone was pRSF-1, which confers kanamycin resistance. The added rDNA target site was composed of rDNA sequence −43 to +21 relative to the initial nick. Template RNA was made with unmodified uridine and had TCARZ 5′ module, chloramphenicol acetyltransferase promoter and ORF, a termination signal for *E. coli* RNAP and 3′ module GeFo 3′ UTR_R4A22. RPE cells, 1 million per treatment, were reverse-transfected in six-well plates with 1.5 µg RNA at a 1/3 molar ratio mRNA/template and 1 µg target plasmid. RNAs and DNA were added together to Lipofectamine 3000; then that mixture was added to cells. Cells were collected 1 d post-transfection and plasmids were separated from chromosomal DNA largely as described^88,89^. Cells were washed twice with Dulbecco’s PBS (Thermo Fisher, catalog no. J67802) and then lysed in the dish by incubation with 400 µl of lysis buffer (10 mM Tris-HCl pH 8.0, 10 mM EDTA, 0.6% SDS) for 5 min at room temperature. Lysates were transferred into 1.5 ml tubes followed by addition of 1/4 volume 5 M NaCl and overnight incubation at 4 °C to precipitate gDNA. Lysates were then spun at 18,000*g* for 30 min and plasmid DNA in the supernatant was purified using PCI followed by a chloroform back-extraction and ethanol precipitation. Pellets were resuspended in 7 µl of nuclease-free water and 1 µl was electroporated into 20 µl of ElectroMAX DH10B competent cells (Thermo Fisher, catalog no. 18290015) following the recommended settings of the manufacturer. Following a 2 h recovery period shaking at 37 °C, 1/30 of the transformation was plated on Luria-Bertani agar plates containing kanamycin and chloramphenicol. Colonies were manually counted and picked at random for full-plasmid nanopore sequencing (Primordium Laboratories).

AB1 files were converted to fastq format with biopython^90^ (v.1.79) and then aligned with minimap2 (refs. ^91,92^) to a reference sequence containing the transgene precisely inserted at the target site. Unmapped portions of reads exceeding 20 nt were aligned again to the reference plasmid (using bwa mem v.0.7.17) to map any duplicated segments. Portions of reads remaining unaligned were then investigated manually using NCBI BLAST. Plasmids with inferred recombination during *E. coli* growth or inverted transgene insertions were excluded from further analysis. To estimate the error rate of transgene sequence insertion, individual plasmid consensus sequences were aligned in a pairwise fashion to the reference plasmid using biopython pairwise2.align.globalms with match score of 2, mismatch penalty of −1, gap opening penalty of −2 and gap extension penalty of −1. From pairwise alignments, the number of substitutions or additional nucleotides was counted. Because homopolymer sequences are a known source of error for full-plasmid sequencing, any changes within homopolymers were excluded from analysis. The error rate reported is the ratio of observed substitutions (1) to the total number of sequenced 3′ UTR bp (13,872). As a control, the same procedure was used to search for substitutions in the plasmid backbone, with none found.

### Statistics and reproducibility

Each experiment described in this paper was repeated with at least one biological replicate, with similar results. This includes all experiments for which a representative gel is shown, as well as bar graphs providing results from triplicate technical assays.

### Reporting summary

Further information on research design is available in the Nature Portfolio Reporting Summary linked to this article.

## Online content

Any methods, additional references, Nature Portfolio reporting summaries, source data, extended data, supplementary information, acknowledgements, peer review information; details of author contributions and competing interests; and statements of data and code availability are available at 10.1038/s41587-024-02137-y.

## Supplementary information

Supplementary Information Supplementary Fig. 1. Uncropped gels for main text Figs. 1b–e and 3c. Fig. 2. Uncropped gels for Extended Data Figs. 1b–d, 3m, 4a, 5b and 9a.

Reporting Summary

Supplementary Table 1 Inventory of plasmid, PCR and oligonucleotide sequences used in this study. Sequencing reads of interest, including extra templates, off targets and tandem insertions are also included.

## Data Availability

Supplementary Table 1 provides construct and oligonucleotide sequences used in this study. WGS data were deposited as SRA BioProject ID PRJNA910950 (ref. ^93^).

## Code availability

Code is available^94^ at 10.5281/zenodo.10439696. This page links to GitHub, where future updates to code will be available.
