# Supplementary material for: Harnessing eukaryotic retroelement proteins for transgene insertion into human safe-harbor loci
Source: Nat Biotechnol. 2024 Feb 20;43(1):42–51. doi: 10.1038/s41587-024-02137-y (PMC11371274; doi:10.1038/s41587-024-02137-y)

nature biotechnology

Article https://doi.org/10.1038/s41587-024-02137-y

Harnessing eukaryotic retroelement proteins for transgene insertion into human safe-harbor loci

In the format provided by the authors and unedited

Supplementary Figure 1

Figure 1b

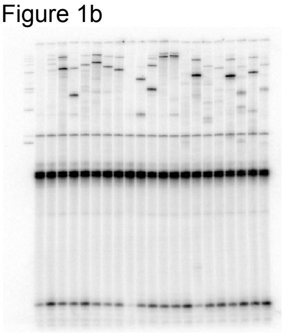

Figure 1c

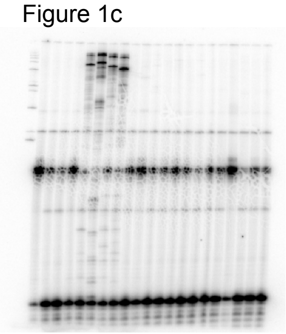

Figure 1d

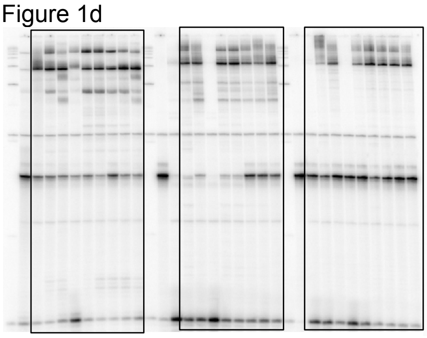

Figure 1e

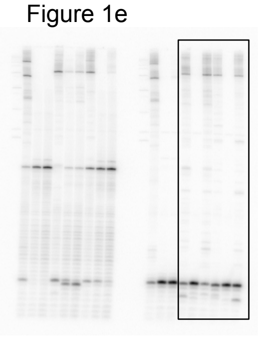

Figure 3c

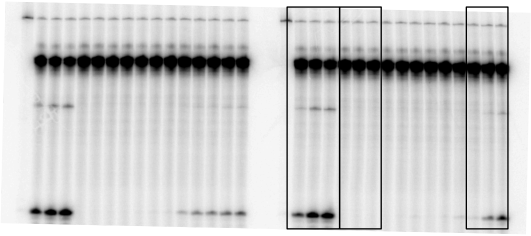

Supplementary Figure 2

ED figure 1b

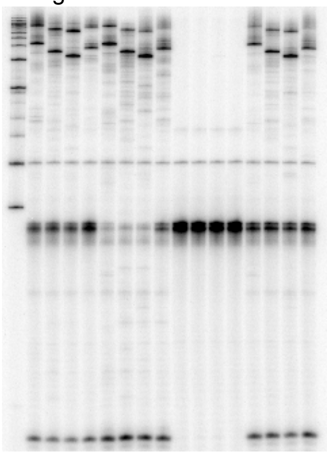

ED figure 1c

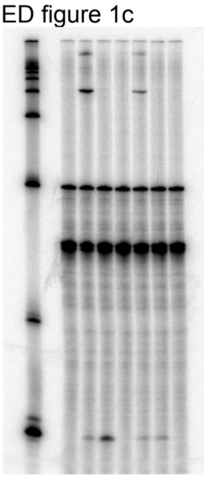

ED figure 1d

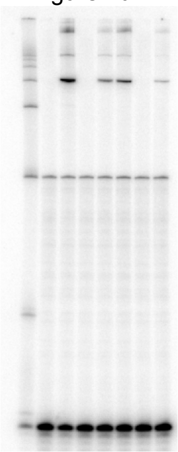

ED figure 3m

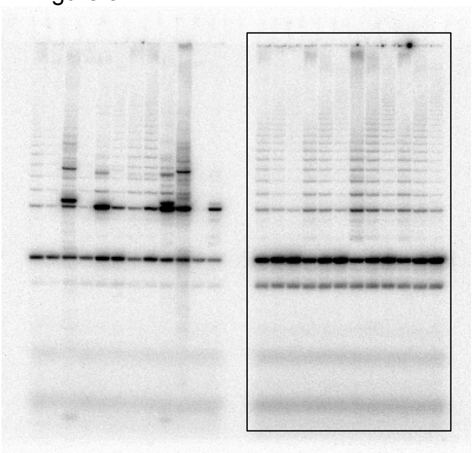

ED figure 4a

Top: p-P53

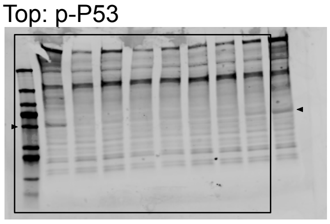

Bottom: tubulin

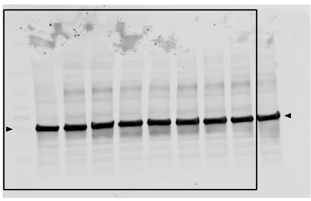

Middle: p-H2A.X

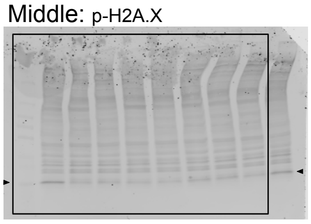

ED figure 5b

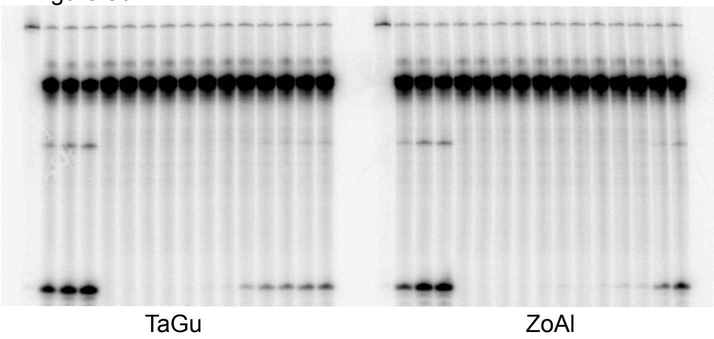

ED figure 9a

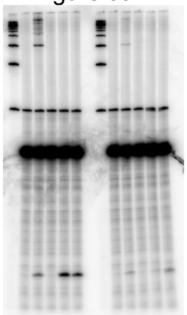

Supplement: Supplementary file 1 — Supplementary Fig. 1. Uncropped gels for main text Figs. 1b–e and 3c. Fig. 2. Uncropped gels for Extended Data Figs. 1b–d, 3m, 4a, 5b and 9a. [file 41587_2024_2137_MOESM1_ESM.pdf]
