# Supplementary material for: Harnessing eukaryotic retroelement proteins for transgene insertion into human safe-harbor loci
Source: Nat Biotechnol. 2024 Feb 20;43(1):42–51. doi: 10.1038/s41587-024-02137-y (PMC11371274; doi:10.1038/s41587-024-02137-y)
Supplement: Supplementary file 2 — Reporting Summary [file 41587_2024_2137_MOESM2_ESM.pdf]

nature portfolio

Corresponding author(s): Kathleen Collins

Last updated by author(s): Jan 8, 2024

Reporting Summary

Nature Portfolio wishes to improve the reproducibility of the work that we publish. This form provides structure for consistency and transparency in reporting. For further information on Nature Portfolio policies, see our Editorial Policies and the Editorial Policy Checklist.

Statistics

For all statistical analyses, confirm that the following items are present in the figure legend, table legend, main text, or Methods section.

n/a	Confirmed	
☐	☒	The exact sample size (*n*) for each experimental group/condition, given as a discrete number and unit of measurement
☐	☒	A statement on whether measurements were taken from distinct samples or whether the same sample was measured repeatedly
☐	☒	The statistical test(s) used AND whether they are one- or two-sided<br>*Only common tests should be described solely by name; describe more complex techniques in the Methods section.*
☒	☐	A description of all covariates tested
☒	☐	A description of any assumptions or corrections, such as tests of normality and adjustment for multiple comparisons
☐	☒	A full description of the statistical parameters including central tendency (e.g. means) or other basic estimates (e.g. regression coefficient) AND variation (e.g. standard deviation) or associated estimates of uncertainty (e.g. confidence intervals)
☐	☒	For null hypothesis testing, the test statistic (e.g. *F*, *t*, *r*) with confidence intervals, effect sizes, degrees of freedom and *P* value noted<br>*Give P values as exact values whenever suitable.*
☒	☐	For Bayesian analysis, information on the choice of priors and Markov chain Monte Carlo settings
☒	☐	For hierarchical and complex designs, identification of the appropriate level for tests and full reporting of outcomes
☒	☐	Estimates of effect sizes (e.g. Cohen's *d*, Pearson's *r*), indicating how they were calculated

Our web collection on statistics for biologists contains articles on many of the points above.

Software and code

Policy information about availability of computer code

Data collection	From "Data availability" section: WGS data was deposited as SRA BioProject ID PRJNA910950. (also noted below)
Data analysis	Code is available at https://doi.org/10.5281/zenodo.10439696. This statement in "Code availability" section of the manuscript is given citation number 93 Zhang, X., Van Treeck, B., Horton, C.A., McIntyre, J.J.R., Palm, S.M., Shumate, J.L. & Collins, K. R2 transgene analysis v1. https://doi.org/10.5281/zenodo.10439696 (2023).

For manuscripts utilizing custom algorithms or software that are central to the research but not yet described in published literature, software must be made available to editors and reviewers. We strongly encourage code deposition in a community repository (e.g. GitHub). See the Nature Portfolio guidelines for submitting code & software for further information.

Data

Policy information about availability of data

All manuscripts must include a data availability statement. This statement should provide the following information, where applicable:

- Accession codes, unique identifiers, or web links for publicly available datasets
- A description of any restrictions on data availability
- For clinical datasets or third party data, please ensure that the statement adheres to our policy

Data availability statement
Supplementary Table 1 provides construct and oligonucleotide sequences used in this study. WGS data was deposited as SRA BioProject ID PRJNA910950.

Research involving human participants, their data, or biological material

Policy information about studies with human participants or human data. See also policy information about sex, gender (identity/presentation), and sexual orientation and race, ethnicity and racism.

Reporting on sex and gender	not applicable
Reporting on race, ethnicity, or other socially relevant groupings	not applicable
Population characteristics	not applicable
Recruitment	not applicable
Ethics oversight	not applicable

Note that full information on the approval of the study protocol must also be provided in the manuscript.

Field-specific reporting

Please select the one below that is the best fit for your research. If you are not sure, read the appropriate sections before making your selection.

☒ Life sciences ☐ Behavioural & social sciences ☐ Ecological, evolutionary & environmental sciences

For a reference copy of the document with all sections, see nature.com/documents/nr-reporting-summary-flat.pdf

Life sciences study design

All studies must disclose on these points even when the disclosure is negative.

Sample size	Sample size was sufficient for quantifications to be informative, supported by statistical significance.
Data exclusions	No data was excluded from the analysis.
Replication	Measurements using technical triplicates for each experiment, with independent experimental replicates. This is stated in Figure legends and Methods.
Randomization	There wasn't an experimental situation amenable to randomization. For example, no siRNA were used where a control siRNA could have randomized sequence.
Blinding	No blinding was performed. We used analysis software to quantify rather than counting anything manually. Quantification methods are included in Methods and flow cytometry gating visuals are provided in an added final Extended Data Figure.

Reporting for specific materials, systems and methods

We require information from authors about some types of materials, experimental systems and methods used in many studies. Here, indicate whether each material, system or method listed is relevant to your study. If you are not sure if a list item applies to your research, read the appropriate section before selecting a response.

Materials & experimental systems

n/a	Involved in the study	
☐	☒	Antibodies
☐	☒	Eukaryotic cell lines
☒	☐	Palaeontology and archaeology
☒	☐	Animals and other organisms
☒	☐	Clinical data
☒	☐	Dual use research of concern
☒	☐	Plants

Methods

n/a	Involved in the study	
☒	☐	ChIP-seq
☐	☒	Flow cytometry
☒	☐	MRI-based neuroimaging

Antibodies

Antibodies used	Listed in the Methods section by name, catalog number, and vendor. For R2 protein: anti-FLAG antibody (Sigma F1804, 1:3000) then Alexa Fluor 680 anti-mouse secondary (Thermo Fisher A21057, 1:2000). For DNA damage assays: rabbit anti-phospho-P53 (Ser15)

(Invitrogen 14H61L24, 1:1000), mouse anti-tubulin (Abcam ab44928, 1:1000), or mouse anti-phospho-histone H2A.X (Ser139) (Invitrogen 6T2311, 1:1000), followed by appropriate secondary, either Alexa Fluor 680 goat anti-rabbit (Invitrogen A21109, 1:2000) or Alexa Fluor Plus 800 goat anti-mouse (Invitrogen A32730, 1:2000).

Validation	Cells lacking R2 protein and cells not subject to DNA damage were used as controls for the two western blotting applications, as described above, respectively.

Eukaryotic cell lines

Policy information about cell lines and Sex and Gender in Research

Cell line source(s)	ATCC and UC Berkeley tissue culture facility. Anyone can order the same cell lines used in this work. hTERT RPE-1 (RPE) and ARPE-19 cells were grown in DMEM/F12 (Gibco) supplemented with 10% fetal bovine serum (FBS) (Seradigm) and 100 g/mL Primocin (InvivoGen). HEK293T, HeLa, IMR-90, MRC-5, and C2C12 cells were grown in DMEM (Gibco) supplemented with 10% FBS. Vero cells were cultivated in DMEM supplemented with 10% FBS and 1% Non-Essential Amino Acid (NEAA, Gibco). All cells were cultured at 37C under 5% CO2 and tested for mycoplasma contamination. Human cell lines were validated by short tandem repeat profiling (Promega, B9510).
Authentication	Human cell lines were validated by short tandem repeat profiling (Promega, B9510). Mouse and monkey cell lines were validated by expected morphology, population doubling time, and positive ddPCR using species-appropriate primer and probe sequences.
Mycoplasma contamination	Tested, negative.
Commonly misidentified lines (See ICLAC register)	none to our knowledge

Flow Cytometry

Plots

Confirm that:

☒ The axis labels state the marker and fluorochrome used (e.g. CD4-FITC).

☒ The axis scales are clearly visible. Include numbers along axes only for bottom left plot of group (a 'group' is an analysis of identical markers).

☐ All plots are contour plots with outliers or pseudocolor plots.

☒ A numerical value for number of cells or percentage (with statistics) is provided.

Methodology

Sample preparation	Cell lines were harvested and fixed as described in Methods.
Instrument	Attune NxT cytometer and Sony SH8000 sorter
Software	Manufacturer's provided software for data collection. FlowJo (10.8.1) used for data analysis and figure preparation. Details of settings are provided in Figure legends and Methods. Gating is shown by quadrants, sectors, or for in some cases of sorting by vertical lines.
Cell population abundance	cell lines were used, without mixtures of cell lines or cell types.
Gating strategy	This differed depending on the application but is given in Figures and Methods and any exceptions are noted in Figure legends. An final Extended Data Figure shows the gating that was used for each quantification. Elsewhere in our instructions the gating figure is requested as Extended Data, whereas in the line below this box it is requested as Supplementary Information.

☒ Tick this box to confirm that a figure exemplifying the gating strategy is provided in the Supplementary Information.
